# Synthesis and In Vitro Anti *Leishmania amazonensis* Biological Screening of Morita-Baylis-Hillman Adducts Prepared from Eugenol, Thymol and Carvacrol

**DOI:** 10.3390/molecules21111483

**Published:** 2016-11-08

**Authors:** Francisco José Seixas Xavier, Klinger Antonio da Franca Rodrigues, Ramon Guerra de Oliveira, Claudio Gabriel Lima Junior, Juliana da Câmara Rocha, Tatjana Souza Lima Keesen, Marcia Rosa de Oliveira, Fábio Pedrosa Lins Silva, Mário Luiz Araújo de Almeida Vasconcellos

**Affiliations:** 1 Laboratório de Síntese Orgânica Medicinal da Paraíba (LASOM-PB), Departamento de Química, Universidade Federal da Paraíba, Campus I, João Pessoa, Paraíba CEP 58059-900, Brazil; seicxhas@hotmail.com (F.J.S.X.); ramonoliveirajp@gmail.com (R.G.d.O.); claudio@quimica.ufpb.br (C.G.L.J.); 2 Programa de Pós-graduação em Biologia Molecular and Departamento de Biologia Molecular, Centro de Ciências Exatas and da Natureza, Universidade Federal da Paraíba, João Pessoa, Paraíba CEP 58059-900, Brazil; klinger.antonio@gmail.com (K.A.d.F.R.); mrosajp@gmail.com (M.R.d.O.); 3 Departamento de Biotecnologia, Universidade Federal da Paraíba, Campus I, João Pessoa, Paraíba CEP 58059-900, Brazil; ju_jurocha@hotmail.com (J.d.C.R.); tat.keesen@cbiotec.ufpb.br (T.S.L.K.)

**Keywords:** Morita-Baylis-Hillman adducts, *Leishmania amazonensis*, eugenol, thymol, carvacrol

## Abstract

Leishmaniasis represents a series of severe neglected tropical diseases caused by protozoa of the genus *Leishmania* and is widely distributed around the world. Here, we present the syntheses of Morita-Baylis-Hillman adducts (MBHAs) prepared from eugenol, thymol and carvacrol, and their bioevaluation against promastigotes of *Leishmania amazonensis*. The new MBHAs are prepared in two steps from essential oils in moderate to good yields and present IC_50_ values in the range of 22.30–4.71 μM. Moreover, the selectivity index to the most potent compound is very high (SIrb > 84.92), far better than that of Glucantime^®^ (SIrb 1.39) and amphotericin B (SIrb = 22.34). Conformational analysis were carried out at the M062X//6-31+G(d,p) level of theory to corroborate a hypothesis about the nitroaromatic bioreduction mechanism.

## 1. Introduction

Leishmaniasis is considered a set of neglected tropical diseases (NTD) caused by protozoa of the genus *Leishmania* and is widely distributed around the world [1]. These diseases are classified into three clinical forms: visceral leishmaniasis (VL), cutaneous leishmaniasis (CL) and mucocutaneous leishmaniasis (MCL). The most abundant species in the Americas, which causes general disfigurement of the face (MCL) and leads to patients withdrawing from social life, are *Leishmania braziliensis* and *Leishmania amazonensis* [2]. Approximately 500,000 new cases of VL are registered each year and are responsible for the deaths of thousands of people. Six countries, one of which is Brazil, account for 90% of deaths related to the disease. The World Health Organization (WHO) estimates that approximately one billion people live in areas at risk, and the 0.9–1.3 million new cases are responsible for the deaths of approximately 30,000 people annually [3]. *Leishmania* spp. is distributed by sandflies, which inject infective promastigotes into the skin of mammalian hosts during a blood meal. To date, there is no leishmaniasis vaccine for humans [4]. Few drug treatment options are available, which are lengthy, toxic, and expensive. In some cases, hospitalization is required, leading to discontinuance of treatment when some improvement is observed in the patient [5]. In addition, *Leishmania* is developing tolerance to the most effective drugs on the market; therefore, the drugs are losing efficiency. Thus, the development of more efficient and cheaper drugs to improve the pharmacological action and the discovery of novel therapeutic targets against trypanosomatids (e.g., *Leishmania* spp.) are major themes of this field [6].

**Scheme 1:**
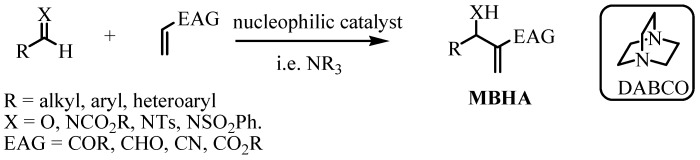
General Morita-Baylis-Hillman reaction. EAG = Electron attractor group.

In the last ten years, our research group has been continuously dedicated to the design, synthesis and biological evaluation of Morita-Baylis-Hillman adducts (MBHAs) as antileishmanial treatments and as treatments against other parasitic NTDs [7]. The Morita-Baylis-Hillman reaction (MBHR, Scheme 1) has been consolidated as an efficient methodology for C-C bond formation [7,8,9]. Polyfunctional MBHA products are obtained in one step by the MBHR by using nucleophilic catalysis (DABCO is the most common catalyst) under metal-free conditions and may be performed in an aqueous solvent mixture or in a solvent-free medium. MBHR is now classified as an important green reaction in organic syntheses [7].

**Scheme 2:**
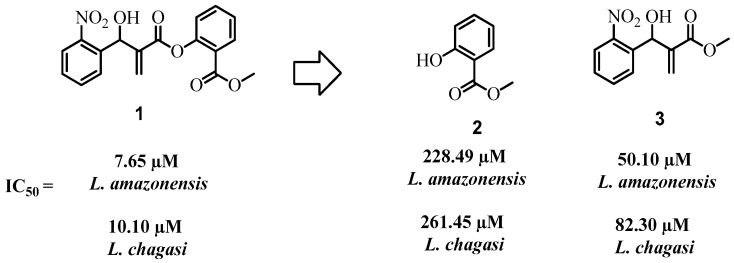
Molecular hybridization between MBHA **3** and methyl salicylate **2** for the design of hybrid **1**.

In 2011, we reported that MBHA **1** (a chalcone-like compound), designed from the molecular hybridization [10] of the analgesic methyl salicylate (**2**) and the MBHA **3** (Scheme 2), presented a lower IC_50_ in a congener series of compounds and was shown to be an efficient molecular hybrid and a promising leishmanicidal drug.

**Figure 1:**
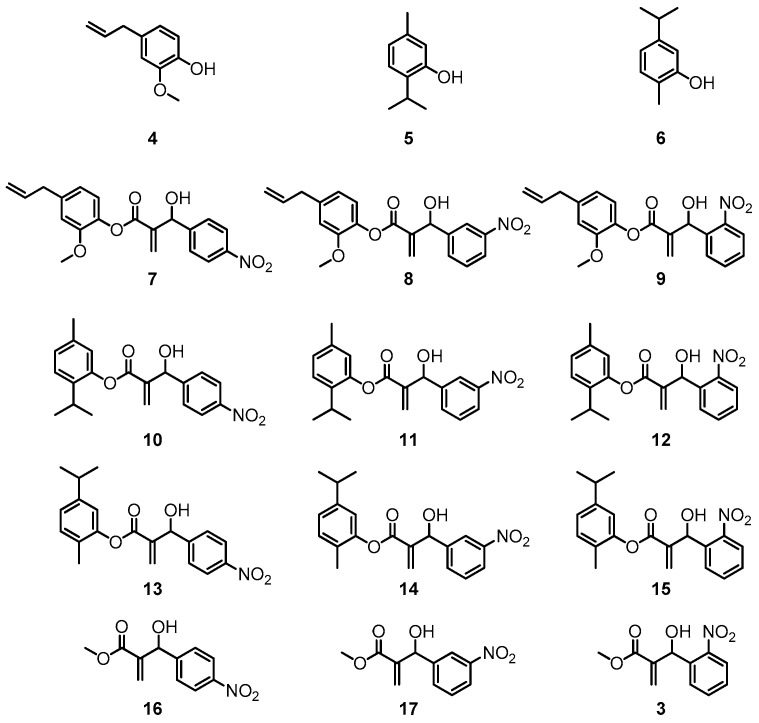
The MBHA hybrids synthetized, biologically evaluated and conformationally studied in this paper and eugenol (**4**), thymol (**5**), and carvacrol (**6**). Compounds **7**–**9** are hybrids derived from eugenol; compounds **10**–**12** are hybrids made from thymol. Compounds **13**–**15** are hybrids of carvacrol. Compounds **16**, **17** and **3** are MBHAs.

In connection with our interest in discovering new compounds with efficient leishmanicidal properties [7,11,12,13,14,15], we present here the design, syntheses, and in vitro leishmanicidal evaluations against *L. amazonensis* of nine new *o-*, *m-* and *p*-nitro MBHA hybrids based on the synergistic biological proprieties of the abundant and inexpensive essential oil ingredients eugenol (**4**), thymol (**5**) and carvacrol (**6**) (Figure 1). It is important to note that eugenol (**4**) is an analgesic with leishmanicidal [16] and several others biological activities [17,18]. Thymol (**5**) and carvacrol (**6**) present analgesic and leishmanicidal activities [19]. In Figure 1, we also present MBHAs **16**, **17** and **3**, which were also biologically evaluated to compare motifs [15]. A conformational DFT study is presented to corroborate our proposed mechanism of the reduction of nitroaryl derivatives concerning these *nitro*-MBHAs [19].

## 2. Results and Discussion

**Scheme 3:**
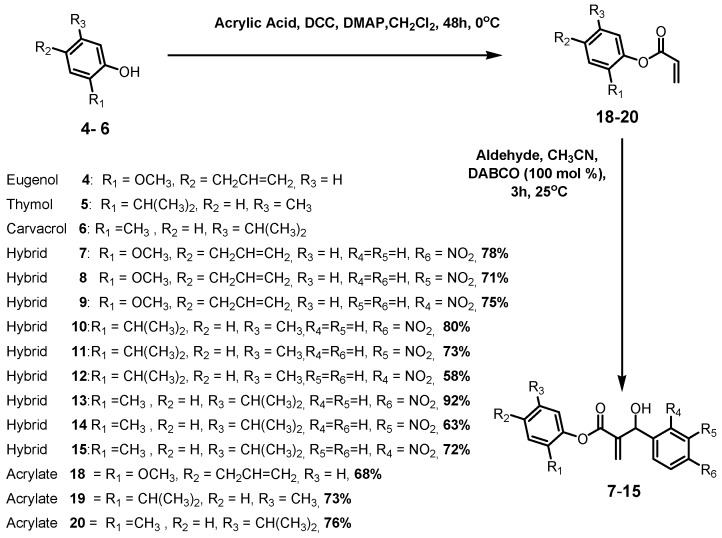
Reagents, conditions, rates, and yields (%) of our preparations of new compounds **7**–**15** and **18**–**20**.

The new acrylates **18**–**20** and hybrids **7**–**15** (Figure 1) were synthesized according to Scheme 3. Eugenol, thymol and carvacrol acrylate **18**–**20** were prepared by the respective reactions with DCC and DMAP (catalytic) at 0 °C in dry dichloromethane (Scheme 3). For the synthesis of hybrids **7**–**15**, acetonitrile was used without drying, however, we observed hydrolysis reactions of the hybrids leading to the products at low yields (<40% yields). To optimize the reaction yields, dry acetonitrile was used as a solvent at room temperature to thus obtain hybrids **7**–**15** in moderate to good yields (Scheme 3).

The antileishmanial activities against promastigotes of *L. amazonensis* of compounds **3**–**17** are presented in Table 1. Some results in this Table should be highlighted here.

**Scheme 4:**
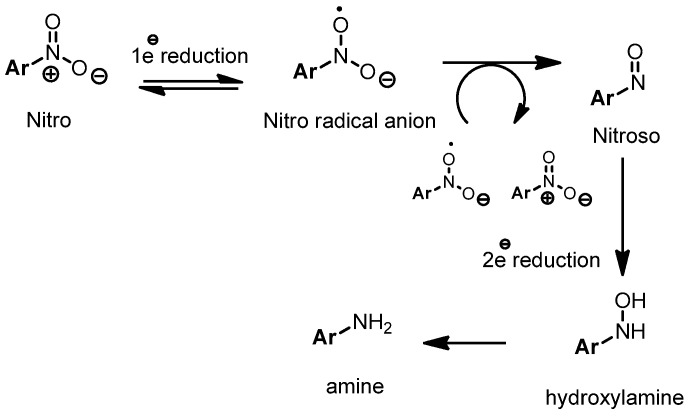
General scheme of the 2e^−^ nitro aromatic reduction (Ar = aromatic group).

We can observe that eugenol (**4**, Entry 1) exhibits greater leishmanicidal activity than the other essential oils thymol (**5**, Entry 5) and carvacrol (**6**, Entry 9). All hybrids derived from eugenol (compounds **7**–**9**, Entries 2–4), thymol (**10**–**12**, Entries 6–8) and carvacrol (**13**–**15**, Entries 11–13) exhibit higher antileishmanial activity than the corresponding essential oils ingredients. The hybrids of eugenol (compounds **7**–**9**, Entries 2–4) showed lower IC_50_ values than the other series. Another very important dataset shown in Table 1, which is in agreement with previously obtained data by our research group [7], indicates that the nitrated MBHAs presenting an *ortho* nitro group on the aromatic ring are more bioactive against promastigotes of *L. amazonensis* than the corresponding *meta* and *para* nitroaryl isomers. Several nitro aromatics are used as anti-infective agents, e.g., drugs to treat parasitic infections. The biological activity of the nitro compounds is connected to a *nitro* group reduction that generates RNO^−•^ or more reduced intermediates (Scheme 4) [19].

**Figure 2:**
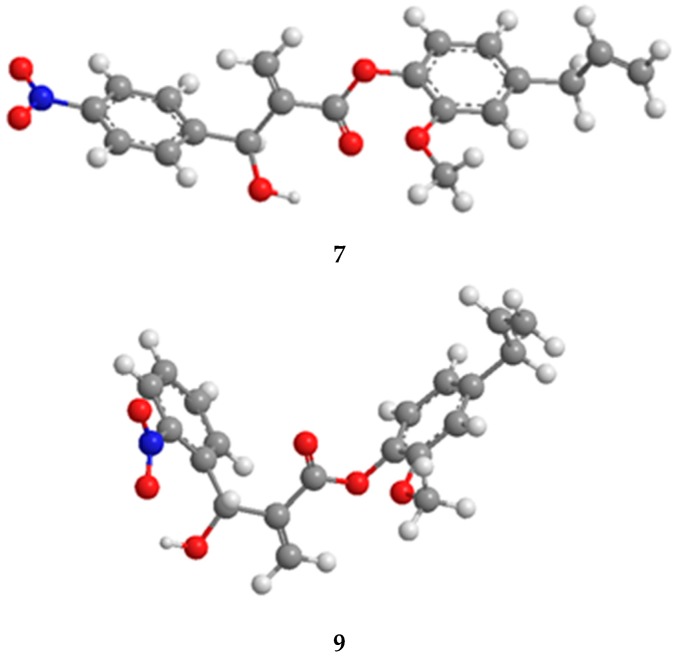
Conformational minima of hybrids **7** and **9** obtained by M062X//6-31+G(d,p) in implicit water simulation as the level of the calculations.

Currently, the exact mechanism of action of MBHAs against *Leishmania* targets is not known. To propose a biological mechanism of action for these new nitroaryl hybrids and to elucidate the reasons behind the higher bioactivity of the *ortho* nitro regioisomer compared to the *meta* and *para* regioisomers, the calculation of the more stable conformation of **7** and **9** in a simulated aqueous medium were performed. These geometries are shown in Figure 2.

In previous electrochemical studies by cyclic voltammetry of the corresponding adducts **16**, **17** and **3** (Figure 1), it was observed that *ortho* aryl **3** is more easily reduced to the corresponding anion radical (ArNO_2_^−•^) of the isomers **16** and **17** [20,21]. It was proposed by QTAIM calculations that the dihedral deviation of the NO_2_ moiety in *ortho* aryl **9** with the aromatic ring is caused by the intramolecular hydrogen bonding (IHB) of the seven-member grouping (HO---O-N-O, IHB = 2.104 Å) [22], which is responsible for the highest electron affinity of this nitrogen. These data are accepted as the reason behind the ease of the biological reduction the *ortho* nitro compounds related to their greater leishmanicidal activity [21]. We can note from Figure 2 that, in the hybrid **7**, the nitro group is nearly coplanar with an aromatic ring (dihedral angle NO_2_ − Ar = 1.7°), which is very different from the observation of hybrid **9** (dihedral angle NO_2_ − Ar = 31.0°). Thus, the radical intermediates from *ortho* MBHA could present higher lifetimes than those of the *para* nitro regioisomer in cytoplasmic medium, increasing the cleavage of the nuclear membrane of protozoa [21].

## 3. Materials and Methods

### 3.1. Experimental Chemistry

#### General

All commercially available reagents and solvents were obtained from the provider Sigma-Aldrich^®^ (St. Louis, MO, USA) and used without further purification. Reactions were monitored by TLC using Silica gel 60 UV254 pre-coated silica gel plates (Macherey-Nagel, Bethlehem, PA, USA) and detection was performed using a UV lamp. Flash column chromatography was performed on 300–400 mesh silica gels. Organic layers were dried over anhydrous MgSO_4_ or Na_2_SO_4_ prior to evaporation using a rotary evaporator. ^1^H-NMR and ^13^C-NMR spectra were recorded using a Mercury Spectra AC 20 spectrometer (200 MHz for ^1^H, 50 MHz for ^13^C, Varian, (Varian, Palo Alto, CA, USA). Chemical shifts were reported relative to internal tetramethylsilane (δ 0.00 ppm) for ^1^H, using CDCl_3_ as the solvent. FTIR spectra were recorded using a model IRPrestige-21 spectrophotometer (Shimadzu, Kyoto, Japan) in KBr pellets. The high-resolution mass spectrometry (HRMS) of new compounds **7**–**15** (Figure 1) was performed using a Q-Tof quadrupole/orthogonal instrument (Waters, Milford, MA, USA) in positive and negative mode.

### 3.2. General Procedure for Esterification of Eugenol (***4***), Thymol (***5***) and Carvacrol (***6***) with Acrylic Acid; Preparation of Compounds ***18***–***20***

Eugenol (**3**, 1.640 g, 10.0 mmol), thymol (**4**, 1.500 g, 10.0 mmol) or carvacrol (**5**, 1.500 g, 10.0 mmol) and dry dichloromethane (10.0 mL) were added in a bottle flask. After homogenization in an ice-bath and magnetic stirring, DCC (2.060 g, 10.0 mmol) and DMAP (0.122 g, 1 mmol) were added. The reaction was monitored by TL chromatography for 48 h. Filtration on a separating funnel was performed. Water was added and the reaction mixture was extracted with dichloromethane (30.0 mL × 2). The organic phase was dried over anhydrous Na_2_SO_4_ and concentrated under reduced pressure using a rotary evaporator. Purification was performed using a chromatographic column containing 30.0 g of flash silica gel, using 100.0 mL of hexane as eluent followed by a mixture of 300 mL ethyl acetate/hexane (30%). The fractions were collected, and the solvent was evaporated from the obtained product using a rotary evaporator. The expected products were colorless, somewhat viscous oils and solid at temperatures below 0 °C.

#### 3.2.1. 4-Allyl-2-methoxyphenyl acrylate (**18**)

68% yield; FTIR KBr: 1745 cm^−1^ (C=O); 1637 cm^−1^ (C=C); 1602 (C=C); 1035 cm^−1^ (C-O-C). ^1^H-NMR δ (ppm): 6.98 (d, *J =* 7.8 Hz, 1H, H-Ar); 6.84–6.73 (m, 2H, H-Ar); 6.60 (dd, *J =* 17.3, 1.6 Hz, 1H, CH_2_=CH); 6.33 (dd, *J =* 17.3, 10.3 Hz, 1H, CH_2_=CH); 6.06–5.84 (m, 2H, CH=CH_2_); 5.17–5.03 (m, 2H, CH_2_=CH); 3.79 (s, 3H, CH_3_OPh); 3.37 (d, *J =* 6.7 Hz, 2H, CH_2_Ph). ^13^C-NMR δ (ppm): 164.28 (C_3_); 150.91 (C_7_); 139.07 (C_9_); 137.67 (C_4_); 137.04 (C_1_); 132.46 (C_10_); 127.64 (C_2_); 122.50 (C_5_); 120.68 (C_6_); 116.18 (C_12_); 112.75 (C_8_); 55.81 (C_13_); 40.10 (C_11_).

#### 3.2.2. 2-Isopropyl-5-methylphenyl acrylate (**19**)

73% yield; FTIR KBr: 1743 cm^−1^ (C=O); 1622 cm^−1^ (C=C). ^1^H-NMR δ (ppm): 7.19 (d, J = 7.9 Hz, 1H, H-Ar); 7.00 (d, *J* = 8,9 Hz, 1H, H-Ar);)6.69 (s, 1H, H-Ar); 6.57 (dd, *J =* 17.2, 1.4 Hz, 1H, CH_2_=CH); 6.33 (dd, *J =* 17.3, 10.3 Hz, 1H, CH=CH_2_); 5.97 (d, *J =* 10.3 Hz, 1H, CH_2_=CH); 2.98–2.75 (m, 1H, HC-(CH_3_)_2_); 2.13 (s, 3H, H_3_C-Ph), 1.23 (d, *J =* 6.9 Hz, 6H, (CH_3_)_2_-CH). ^13^C-NMR δ (ppm): 164.41 (C3); 149.12 (C6); 148.10 (C_4_); 132.43 (C_1_); 130.93 (C_8_); 127.86 (C_2_); 127.23 (C_9_); 124.24 (C_7_); 119.73 (C_5_); 33.60, (C_11_); 23.95 (C_12/12′_); 15.80 (C_10_).

#### 3.2.3. 5-Isopropyl-2-methylphenyl acrylate (**20**)

76% yield; FTIR KBr: 1743 cm^−1^ (C=O); 1635 cm^−1^ (C=C); 1622 cm^−1^ (C=C). ^1^H-NMR δ (ppm): 7.08 (d, *J* = 7.8 Hz, 1H, H-Ar); 6.97 (d, *J* = 7.8 Hz, 1H, H-Ar);) 6.69 (s, 1H, H-Ar); 6.57 (dd, *J =* 17.2, 1.4 Hz, 1H, CH_2_=CH); 6.33 (dd, *J =* 17.3, 10.3 Hz, 1H, CH=CH_2_); 5.97 (d, *J =* 10.3 Hz, 1H, CH_2_=CH); 2.98–2.75 (m, 1H, HC-(CH_3_)_2_); 2.13 (s, 3H, H_3_C-Ph), 1.23 (d, *J =* 6.9 Hz, 6H, (CH_3_)_2_-CH). ^13^C-NMR δ (ppm): 164.41 (C_3_); 149.12 (C_6_); 148.10 (C_4_); 132.43 (C_1_); 130.93 (C_8_); 127.86 (C_2_); 127.23 (C_9_); 124.24 (C_7_); 119.73 (C_5_); 33.60, (C_11_); 23.95 (C_12e12′_); 15.80 (C_10_).

### 3.3. General Synthesis of the MBHAs ***16***, ***17*** and ***3***

The compounds **16**, **17** and **3** synthesized in this work are not new and were characterized using ^1^H-NMR and ^13^C-NMR for comparison with the compounds described in the literature [14]. Reactions were carried out using the corresponding aldehydes (1 mmol), methyl acrylate (0.5 mL) and of DABCO (1 mmol) at 0 °C. After some time, the reaction media was directly filtered through silica gel using EtOAc-hexane (2:8) as the solvent, and the reaction products were concentrated under reduced pressure. The products were then ready for biological evaluations without the need of further purification.

#### 3.3.1. 2-[Hydroxy(4-nitrophenyl)methyl]propanoate (**16**)

85% yield; ^1^H-NMR δ (ppm): d, 8.16 (d, 2H, *J =* 8.8 Hz), 7.54 (d, 2H, *J =* 8.4 Hz), 6.36 (s, 1H), 5.86 (s, 1H), 5.60 (d, *J =* 5.8 Hz, 1H), 3.71 (s, 3H), 3.41 (d, *J =* 5.8 Hz, 1H). ^13^C-NMR δ (ppm): 52.2 (1C), 72.6 (1C), 123.5 (1C), 137.2 (1C), 127.3 (1C), 140.8 (1C), 147.3 (1C), 148.5 (1C), 166.3 (1C).

#### 3.3.2. 2-[Hydroxy(3-nitrophenyl)methyl]propanoate (**17**)

98% yield; ^1^H-NMR δ (ppm): d, 8.22 (t, *J =* 1.8 Hz, 1H); 8.08–8.13 (m, 1H); 7.69–7.73 (m, 1H); 7.50 (t, *J =* 8 Hz, 1H); 6.38 (s, 1H); 5.89 (s, 1H); 5.60 (d, *J =* 4.4 Hz, 1H); 3.70 (s, 3H); 3.46 (d, *J =* 5.4 Hz, 1H). ^13^C-NMR δ (ppm): 52.2 (1C), 72.5 (1C), 121.5 (1C), 122.7 (1C), 127.2 (1C), 129.3 (1C), 132.7 (1C), 140.9 (1C), 143.5 (1C), 148.2 (1C), 166.3(1C).

#### 3.3.3. 2-[Hydroxy(2-nitrophenyl)methyl]propanoate (**3**)

92% yield; ^1^H-NMR δ (ppm): d, 7.92 (dd, *J =* 8.2/1.2 Hz, 1H); 7.73 (dd, *J =* 7.8/1.8 Hz, 1H); 7.62 (ddd, *J =* 7.8/1.2/1.0 Hz, 1H); 7.43 (ddd, *J =* 8.0/1.6/1.6 Hz, 1H); 6.38 (s, 1H); 5.89 (d, 1H); 5.60 (d, *J =* 4.4 Hz, 1 Hz); 3.70 (s, 3H); 3.60 (d, *J =* 5.4 Hz, 1H). ^13^C-NMR δ (ppm): 52.20 (1C), 67.56 (1C), 124.55 (1C), 126.48 (1C), 128.67 (C), 128.67 (C) 133.47 (1C), 136.05 (1C), 140.65 (1C), 148.32 (1C), 166.39(1C).

### 3.4. General Synthesis of the Hybrids ***7***–***15***

To a 25.0 mL flask, acrylate **18**, **19** or **20** (0.5 mmol), the corresponding nitro-substituted aldehyde (0.6 mmol), and dry acetonitrile (1.0 mL) were added. To the resulting solution DABCO (0.5 mmol) was added. The reaction mixture was stirred at room temperature and monitored by TLC for 3 h. The purified product was obtained by using 6.0 g of silica and 10.0 mL of acetonitrile to form a dispersion and added to a chromatographic column containing 30.0 g of flash silica gel. A mixture of 300 mL ethyl acetate/hexane 5% followed by 200 mL 15% acetate/hexane was used as an eluent. The fractions were separated and transferred to a rotary evaporator. The respective products were yellowish viscous oils at ambient temperature. All spectra are available in the Supplementary information.

#### 3.4.1. 4-Allyl-2-methoxyphenyl 2-(hydroxy(4-nitrophenyl)methyl)acrylate (**7**)

78% yield; FTIR: 3450 cm^−1^ (O-H); 1735 cm^−1^ (C=O); 1637 cm^−1^ (C=C); 1602 cm^−1^ (C=C); 1531 and 1350 cm^−1^ (R-NO_2_), 1035 cm^−1^ (C-O-C). ^1^H-NMR δ (ppm): 8.21 (d, *J =* 8.8 Hz, 2H, H-Ar); 7.63 (d, *J =* 8.8 Hz, 2H, H-Ar); 6.95–6.89 (m, 1H, H-Ar); 6.81–6.73 (m, 2H, H-Ar); 6.64 (s, 1H, H-C-OH); 6.02 (d, *J =* 7.9 Hz, 1H, CH_2_=C); 5.96–5.70 (m, 2H, CH=CH_2_ and CH_2_=C); 5.16–5.03 (m, 2H, CH_2_=CH); 3.72 (s, 3H, CH_3_OPh); 3.40 (t, *J =* 13.3 Hz, 3H, CH_2_Ph and HO-C). ^13^C-NMR δ (ppm): 168.81 (C_8_); 155.16 (C_14_); 153.19 (C_4_); 152.16 (C_3_); 145.44 (C_6_); 144.22 (C_12_); 141.95 (C_9_); 141.56 (C_16_); 133.51 (C_7_); 132.24 (C1e1′); 128.29 (C2e2′); 127.01 (C_10_); 125.46 (C_11_); 121.03 (C_17_); 117.37 (C_13_); 77.33 (C_5_); 60.45 (C_18_); 44.78 (C_15_). HRMS (TOF MS ES−) calculated for C_20_H_19_NO_6_: 369.3700 [M + H]^+^; found: 370.1298.

#### 3.4.2. 4-Allyl-2-methoxyphenyl-2-(hydroxy(3-nitrophenyl)methyl)acrylate (**8**)

71% yield; FTIR: 3450 cm^−1^ (O-H); 1735 cm^−1^ (C=O); 1637 cm^−1^ (C=C); 1602 cm^−1^ (C=C); 1531 and 1350 cm^−1^ (R-NO_2_), 1035 cm^−1^ (C-O-C). ^1^H-NMR δ (ppm): 8.33 (s, 1H, H-Ar); 8.17 (d, *J =* 7,0 Hz, 1H, H-Ar); 7.80 (d, *J =* 8.2 Hz, 1H, H-Ar); 7.54 (t, *J =* 7.9 Hz, 1H, H-Ar); 6.93 (d, *J =* 8.6 Hz, 1H, H-Ar); 6.75 (d, *J =* 7.3 Hz, 2H, H-Ar); 6.66 (s, 1H, H-C-OH); 6.03 (s, 1H, CH_2_=C); 5.75–5.95 (m, 2H, CH=CH_2_ and CH_2_=C); 5.14–5.04 (m, 2H, CH_2_=CH); 3.72 (s, 3H, CH_3_OPh); 3.36 (d, *J =* 6.7 Hz, 3H, CH_2_Ph and HO-C). ^13^C-NMR δ (ppm): 164.84 (C_10_); 151.22 (C_16_); 149.09 (C_4_); 144.23 (C_6_); 141.43 (C_9_); 140.21 (C_14_); 138.02 (C_11_); 137.63 (C_18_); 133.62 (C_1_); 130.09 (C_2_); 129.58 (C_8_); 123.55 (C_5_); 123.06 (C_3_); 122.45 (C_12_); 121.48 (C_13_); 117.02 (C_19_); 113.40 (C_15_); 73.35 (C_7_); 56.53 (C_20_); 40.83 (C_17_). HRMS (TOF MS ES−) calculated for C_20_H_19_NO_6_: 369.3700 [M + H]^+^; found: 370.1390.

#### 3.4.3. 4-Allyl-2-methoxyphenyl 2-(hydroxy(2-nitrophenyl)methyl)acrylate (**9**)

75% yield; FTIR: 3458 cm^−1^ (O-H); 1737 cm^−1^ (C=O); 1637 cm^−1^ (C=C); 1604 cm^−1^ (C=C); 1527 and 1348 cm^−1^ (R-NO_2_), 1033 cm^−1^ (C-O-C). ^1^H-NMR δ (ppm): 7.96–8.06 (m, 1H, H-Ar); 7.83 (d, *J =* 7.8 Hz, 1H, H-Ar); 7.67 (dd, *J =* 8.3, 4.6 Hz, 1H, H-Ar); 7.50 (d, *J =* 6.7 Hz, 1H, H-Ar); 6.93 (d, *J =* 8.6 Hz, 1H, H-Ar); 6.79–6.70 (m, 2H, H-Ar); 6.61 (s, 1H, H-C-OH); 6.31 (s, 1H, CH_2_=C); 6.05–5.81 (m, 2H, CH=CH_2_ and CH_2_=C); 5.16–5.01 (m, 2H, CH_2_=CH); 3.73 (s, 4H, CH_3_OPh and HO-C); 3.36 (d, *J =* 6.7 Hz, 2H, CH_2_Ph). ^13^C-NMR δ (ppm): 164.12 (C_10_); 150.52 (C_16_); 148.32 (C_5_); 140.25 (C_9_); 139.21 (C_14_); 137.43 (C_6_); 136.92 (C_18_); 135.99 (C_11_); 133.50 (C_2_); 129.09 (C_3_); 128.72 (C_1_); 128.06 (C_8_); 124.67 (C_4_); 122.34 (C_12_); 120.65 (C_13_); 116.18 (C_19_); 112.61 (C_15_); 67.88 (C_7_); 55.72 (C_20_); 40.04 (C_17_). HRMS (TOF MS ES−) calculated for C_20_H_19_NO_6_: 369.3700 [M + H]^+^; found: 370.1298.

#### 3.4.4. 2-Isopropyl-5-methylphenyl 2-(hydroxy(4-nitrophenyl)methyl)acrylate (**10**)

80% yield; FTIR: 3485 cm^−1^ (O-H); 1732 cm^−1^ (C=O); 1606 cm^−1^ (C=C); 1523 and 1348 cm^−1^ (R-NO_2_). ^1^H-NMR δ (ppm): 8.20 (d, *J =* 8.8 Hz, 2H, H-Ar); 7.63 (d, *J =* 8.6 Hz, 2H, H-Ar); 7.06 (d, *J* = 7.8 Hz, 2H, H-Ar); 6.78 (d, *J =* 7.3 Hz, 1H, H-Ar); 6.67 (s, 1H, H-C-OH); 6.12 (s, 1H, CH_2_=C); 5.75 (d, *J =* 4.8 Hz, 1H, CH_2_=C); 3.50 (d, *J =* 4.9 Hz, 1H, HO-C); 2.85 (hept, *J =* 6.8 Hz, 1H, HC-(CH_3_)_2_); 1.95 (s, 3H, H_3_C-Ph); 1.20 (d, *J =* 6.9 Hz, 6H, (CH_3_)_2_-CH). ^13^C-NMR δ (ppm): 164.24 (C_8_); 148.63 (C_4_); 148.27 (C_9_); 147.48 (C_3_); 140.93 (C_11_); 131.01 (C_13_); 128.23 (C_6_); 128.16 (C_7_); 127.56 (C_2/2′_); 126.95 (C_14_); 124.49 (C_12_); 123.63 (C_1/1′_); 119.46 (C_10_); 72.36 (C_5_); 33.50 (C_16_); 23.85 (C_17/17′_); 15.57 (C_15_). HRMS (TOF MS ES−) calculated for C_20_H_21_NO_5_: 355.1400 [M − H]^−^; found: 354.1426.

#### 3.4.5. 2-Isopropyl-5-methylphenyl 2-(hydroxy(3-nitrophenyl)methyl)acrylate (**11**)

73% yield; FTIR: 3493 cm^−1^ (O-H); 1730 cm^−1^ (C=O); 1637 cm^−1^ (C=C); 1622 cm^−1^ (C=C); 1531 and 1350 cm^−1^ (R-NO_2_).^1^H-NMR δ (ppm): 8.30 (s, 1H, H-Ar); 8.15 (dd, *J =* 8.2, 1.1 Hz, 1H, H-Ar); 7.79 (d, *J =* 7.7 Hz, 1H, H-Ar); 7.53 (t, *J =* 7.9 Hz, 1H, H-Ar); 7.06 (dd, *J =* 7.8 Hz, 2H, H-Ar); 6.79 (s, 1H, H-Ar); 6.68 (s, 1H, H-C-OH); 6.13 (s, 1H, CH_2_=C); 5.74 (d, *J =* 5.2 Hz, 1H, CH_2_=C); 3.36 (d, *J =* 5.6 Hz, 1H, HO-C), 2.76–2.60 (m, 1H, HC-(CH_3_)_2_); 1.95 (s, 3H, H_3_C-Ph); 1.20 (d, *J =* 6.9 Hz, 6H, (CH_3_)_2_-CH). ^13^C-NMR δ (ppm): 164.29 (C_10_); 148.69 (C_11_); 148.29 (C_4_); 143.56 (C_13_); 140.81 (C_6_); 132.90 (C_1_); 131.01 (C_2_); 129.44 (C_15_); 128.36 (C_8_); 128.28 (C_9_); 126.99 (C_16_); 124.51 (C_5_); 122.89 (C_3_); 121.65 (C_14_); 119.49 (C_12_); 72.17 (C_7_); 33.51 (C_17_); 23.87 (C_18/18′_); 15.60 (C_19_). HRMS (TOF MS ES−) calculated for C_20_H_21_NO_5_: 355.1400 [M − H]^−^; found: 354.1154.

#### 3.4.6. 2-Isopropyl-5-methylphenyl 2-(hydroxy(2-nitrophenyl)methyl)acrylate (**12**)

58% yield; FTIR: 3450 cm^−1^ (O-H); 1734 cm^−1^ (C=O); 1620 cm^−1^ (C=C); 1527 and 1348 cm^−1^ (R-NO_2_). ^1^H- NMR δ (ppm): 7.87 (d, *J =* 7.5 Hz, 2H, H-Ar); 7.62 (t, *J =* 7.5 Hz, 1H, H-Ar); 7.42 (t, *J =* 7.7 Hz, 1H, H-Ar); 7.03 (d, *J =* 7.8 Hz, 2H, H-Ar); 6.81 (s, 1H, H-Ar); 6.58 (s, 1H, H-C-OH); 6.30 (s, 1H, CH_2_=C); 5.86 (s, 1H, CH_2_=C); 3.82 (s, 1H, HO-C); 2.95–2.74 (m, 1H, HC-(CH_3_)_2_); 1.96 (s, 3H, H_3_C-Ph); 1.19 (d, *J =* 6.9 Hz, 6H, (CH_3_)_2_-CH). ^13^C-NMR δ (ppm): 164.36 (C_10_); 148.92 (C_11_); 148.12 (C_5_); 140.83 (C_13_); 136.39 (C_8_); 133.61 (C_2_); 130.93 (C_15_); 128.96 (C_3_); 128.79 (C_1_); 127.70 (C_6_); 127.66 (C_9_); 127.18 (C_16_); 124.78 (C_4_); 124.32 (C_14_); 119.61 (C_12_); 67.54 (C_7_); 33.53 (C_17_); 23.89 (C_18/18′_); 15.55 (C_19_). HRMS (TOF MS ES−) calculated for C_20_H_21_NO_5_: 355.1400 [M − H]^−^; found: 354.1335.

#### 3.4.7. 5-Isopropyl-2-methylphenyl 2-(hydroxy(4-nitrophenyl)methyl)acrylate (**13**)

92% yield; FTIR: 3510 cm^−1^ (O-H); 1728 cm^−1^ (C=O); 1624 cm^−1^ (C=C); 1606 cm^−1^ (C=C); 1521 and 1348 cm^−1^ (R-NO_2_). ^1^H-NMR δ (ppm): 8.20 (d, *J =* 8.8 Hz, 2H, H-Ar); 7.63 (d, *J =* 8.6 Hz, 2H, H-Ar); 7.06 (dd, *J =* 21.4, 7.8 Hz, 2H, H-Ar); 6.78 (d, *J =* 1.2 Hz, 1H, H-Ar); 6.67 (s, 1H, H-C-OH); 6.12 (s, 1H, CH_2_=C); 5.75 (d, *J =* 4.8 Hz, 1H, CH_2_=C); 3.50 (d, *J =* 4.9 Hz, 1H, HO-C); 2.85 (hept, *J =* 6.8 Hz, 1H, HC-(CH_3_)_2_); 1.95 (s, 3H, H_3_C-Ph); 1.20 (d, *J =* 6.9 Hz, 6H, (CH_3_)_2_-CH). ^13^C-NMR δ (ppm): 164.24 (C_8_); 148.63 (C_4_); 148.27 (C_9_); 147.48 (C_3_); 140.93 (C_11_); 131.01 (C_13_); 128.23 (C_6_); 128.16 (C_7_); 127.56 (C_2e2′_); 126.95 (C_14_); 124.49 (C_12_); 123.63 (C_1e1′_); 119.46 (C_10_); 72.36 (C_5_); 33.50 (C_16_); 23.85 (C_17e17′_); 15.57 (C_15_). HRMS (TOF MS ES−) calculated for C_20_H_21_NO_5_: 355.1400 [M − H]^−^; found: 354.1335.

#### 3.4.8. 5-Isopropyl-2-methylphenyl 2-(hydroxy(3-nitrophenyl)methyl)acrylate (**14**)

63% yield; FTIR: 3450 cm^−1^ (O-H); 1735 cm^−1^ (C=O); 1637 cm^−1^ (C=C); 1602 cm^−1^ (C=C); 1531 and 1350 cm^−1^ (R-NO_2_). ^1^H-NMR δ (ppm): 8.30 (s, 1H, H-Ar); 8.14 (d, *J =* 8.2 Hz, 1H, H-Ar); 7.79 (d, *J =* 7.7 Hz, 1H, H-Ar); 7.53 (t, *J =* 7.9 Hz, 1H, H-Ar); 7.08 (d, *J =*7.8 Hz, 2H, H-Ar); 6.79 (s, 1H, H-Ar); 6.68 (s, 1H, H-C-OH); 6.13 (s, 1H, CH_2_=C); 5.74 (d, *J =* 5.2 Hz, 1H, CH_2_=C); 3.36 (d, *J =* 5.6 Hz, 1H, HO-C), 2.97–2.71 (m, 1H, HC-(CH_3_)_2_); 1.95 (s, 3H, H_3_C-Ph); 1.20 (d, *J =* 6.9 Hz, 6H, (CH_3_)_2_-CH). ^13^C-NMR δ (ppm): 164.29 (C_10_); 148.69 (C_11_); 148.29 (C_4_); 143.56 (C_13_); 140.81 (C_6_); 132.90 (C_1_); 131.01 (C_2_); 129.44 (C_15_); 128.36 (C_8_); 128.28 (C_9_); 126.99 (C_16_); 124.51 (C_5_); 122.89 (C_3_); 121.65 (C_14_); 119.49 (C_12_); 72.17 (C_7_); 33.51 (C_17_); 23.87 (C_18/18′_); 15.60 (C_19_). HRMS (TOF MS ES−) calculated for C_20_H_21_NO_5_: 355.1400 [M − H]^−^; found: 354.1154.

#### 3.4.9. 5-Isopropyl-2-methylphenyl 2-(hydroxy(2-nitrophenyl)methyl)acrylate (**15**)

72% yield; FTIR: 3489 cm^−1^ (O-H); 1734 cm^−1^ (C=O); 1624 cm^−1^ (C=C); 1527 and 1348 cm^−1^ (R-NO_2_). ^1^H- NMR δ (ppm): 7.86 (d, *J =* 7.5 Hz, 2H, H-Ar); 7.62 (t, *J =* 7.5 Hz, 1H, H-Ar); 7.42 (t, *J =* 7.7 Hz, 1H, H-Ar); 7.03 (d, *J =* 7.9 Hz, 2H, H-Ar); 6.81 (s, 1H, H-Ar); 6.58 (s, 1H, H-C-OH); 6.30 (s, 1H, CH_2_=C); 5.86 (s, 1H, CH_2_=C); 3.82 (s, 1H, HO-C); 2.99–2.67 (m, 1H, HC-(CH_3_)_2_); 1.96 (s, 3H, H_3_C-Ph); 1.19 (d, *J =* 6.9 Hz, 6H, (CH_3_)_2_-CH). ^13^C-NMR δ (ppm): 164.36 (C_10_); 148.92 (C_11_); 148.12 (C_5_); 140.83 (C_13_); 136.39 (C_8_); 133.61 (C_2_); 130.93 (C_15_); 128.96 (C_3_); 128.79 (C_1_); 127.70 (C_6_); 127.66 (C_9_); 127.18 (C_16_); 124.78 (C_4_); 124.32 (C_14_); 119.61 (C_12_); 67.54 (C_7_); 33.53 (C_17_); 23.89 (C_18/18′_); 15.55 (C_19_). HRMS (TOF MS ES) calculated for C_20_H_21_NO_5_: 355.1400 [M − H]^−^; found: 354.1516.

### 3.5. Biology

The promastigote viabilities were determined by the ability of living cells to reduce the yellow MTT (3-(4,5-dimethylthiazol-2-yl)-2,5-diphenyltetrazolium bromide, a tetrazole) to purple formazan [23]. Promastigote forms of *Leishmania amazonensis* (IFLA/BR/67/PH8) in the log phase of growth were incubated at 25 °C in 96-well cellular culture plates (TPP, Trasadingen, Switzerland) with 1 × 10^5^ cells/well in 100 μL Schneider’s Drosophila medium supplemented with 20% of FBS in the presence or absence of different concentrations of compounds **4**–**15**. The growth of promastigotes forms was evaluated simultaneously in the presence of Glucantime^®^, as reference drug. After 72 h of incubation, 10 mL of a 5 mg·mL^−1^ MTT solution was added. After 4 h of incubation at 25 °C, the formed product formazan was dissolved in 50 mL of sodium dodecyl sulfate (SDS) at 10% for 24 h, and the absorbance was measured by spectrophotometry at a 545 nm wavelength. The percentage viability was calculated from the ratio of OD readings in the wells with compounds versus the wells without compounds × 100. The concentration that inhibited 50% of growth (IC_50_) was determined by regression analysis using the SPSS 8.0 software for Windows (IBM, New York, NY, USA). All experiments were performed at least three times, and each experiment was performed in triplicate [16]. Haemolytic assays (HC_50_ > 400 μM) showed that the **4**, **7**, **8**, **9**, **16**, **17** and **3** exhibit low toxicity towards human red blood cells, unlike amphotericin B (HC_50_ = 11.61 μM Table 1) [24].

### 3.6. Conformational Analysis

**Figure 3:**
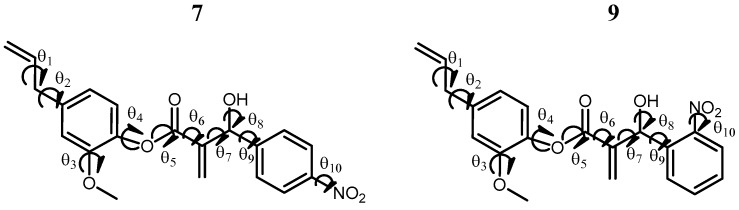
Computational methodology used to minimize the new hybrids **7** and **9**. Optimized geometries (Cartesian coordinates) to **7** and **9** are available in the Supplementary information.

The relaxed potential energy surface scan (RPESS) was performed by taking particular care of the dihedral angles θ_1_–θ_10_, which are illustrated in Figure 3. These calculations were executed by using the semi-empiric PM6 level with angle variations from 0° to 360° in intervals of 10°. Next, the minimum conformation was fully optimized by M062x//6-31+G(d,p) as the level of the calculations without any symmetry constraint and with all geometries modeled at a minimum of potential energy because no imaginary frequencies were found. The minima of hybrids **7** and **9** were optimized in water implicit simulations using the CPCM methodology by GAUSSIAN09L [25].

## 4. Conclusions

The nine new molecular hybrids designed in this study were synthesized in good yields in two steps from eugenol, thymol and carvacrol and presented IC_50_ values in the range of 22.30–4.71 μM to promastigotes of *Leishmania amazonensis*. Besides, the most active hybrid **9** does not exhibit significant toxicity in red blood cell studies (SIrb > 84.92) which suggests the possibility of an advancement in CL and MCL treatment. The experimental studies of bioactivity and conformational DFT calculations presented here are in full agreement with our first mechanistic proposal whereby the biological activity of nitro compounds is connected to nitro group reduction that generates RNO^−•^. Separation of the enantiomers of the new compound **9** by kinetic resolution catalyzed by lipase B from *Candida antarctica* [26] and performing in vivo studies will continue this present work.

## Figures and Tables

**Table 1:** The antileishmanial activities against promastigotes of *L. amazonensis*. Values represent the average IC_50_ calculated in three independent experiments ± standard error of the mean. For this calculation, we used the probabilistic regression models with SPSS 13.0 software. SIrb (selectivity index on red blood cell) = HC_50_/IC_50_.

Entry	Compounds	IC_50_ (μM)	HC_50_ (μM)	SIrb	miLogP
1	**4**	77.15 ± 1.77	>400	>5.18	2.10
2	**7**	8.75 ± 0.44	>400	>45.71	3.05
3	**8**	10.49 ± 0.52	>400	>38.13	3.02
4	**9**	4.71 ± 0.24	>400	>84.92	3.00
5	**5**	115.12 ± 5.77	-	-	3.34
6	**10**	11.40 ± 0.57	-	-	4.05
7	**11**	10.56 ± 0.53	-	-	4.03
8	**12**	5.91 ± 0.30	-	-	4.00
9	**6**	138.11 ± 6.91	-	-	3.81
11	**13**	18.08 ± 0.90	-	-	4.52
12	**14**	22.30 ± 1.12	-	-	4.50
13	**15**	13.60 ± 0.68	-	-	4.47
14	**16**	15.77 ± 0.79	>400	>25.36	1.53
15	**17**	22.38 ± 1.12	>400	>17.87	1.50
16	**3**	12.06 ± 0.60	>400	>33.16	1.48
17	Glucantime^®^	1633.49 ± 437.29	1175.02	1.39	−2.68
18	AmphotericinB	0.52 ± 0.03	11.61	22.34	−2.49

## Supplementary Materials

Supplementary information related to this article is available at www.mdpi.com/1420-3049/21/11/1483/s1.
