# Supplementary material for: Synthesis and In Vitro Anti Leishmania amazonensis Biological Screening of Morita-Baylis-Hillman Adducts Prepared from Eugenol, Thymol and Carvacrol
Source: Molecules. 2016 Nov 8;21(11):1483. doi: 10.3390/molecules21111483 (PMC6274563; doi:10.3390/molecules21111483)
Supplement: Supplementary file 1 [file molecules-21-01483-s001.pdf]

Supplementary Materials: Synthesis and *In Vitro* Anti *Leishmania amazonensis* Biological Screening of Morita-Baylis-Hillman Adducts Prepared from Eugenol, Thymol and Carvacrol

Francisco José Seixas Xavier, Klinger Antonio da Franca Rodrigues, Ramon Guerra de Oliveira, Claudio Gabriel Lima Junior, Juliana da Câmara Rocha, Tatjana Souza Lima Keesen, Marcia Rosa de Oliveira, Fábio Pedrosa Lins Silva and Mário Luiz Araújo de Almeida Vasconcellos

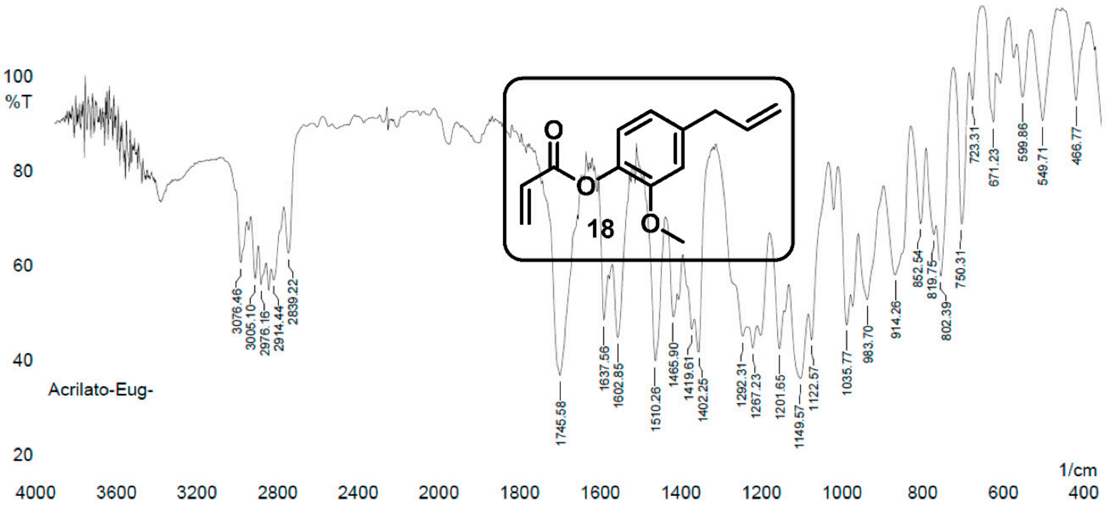

Figure S1. FTIR (KBr) spectrum of 4-allyl-2-methoxyphenyl acrylate **18**.

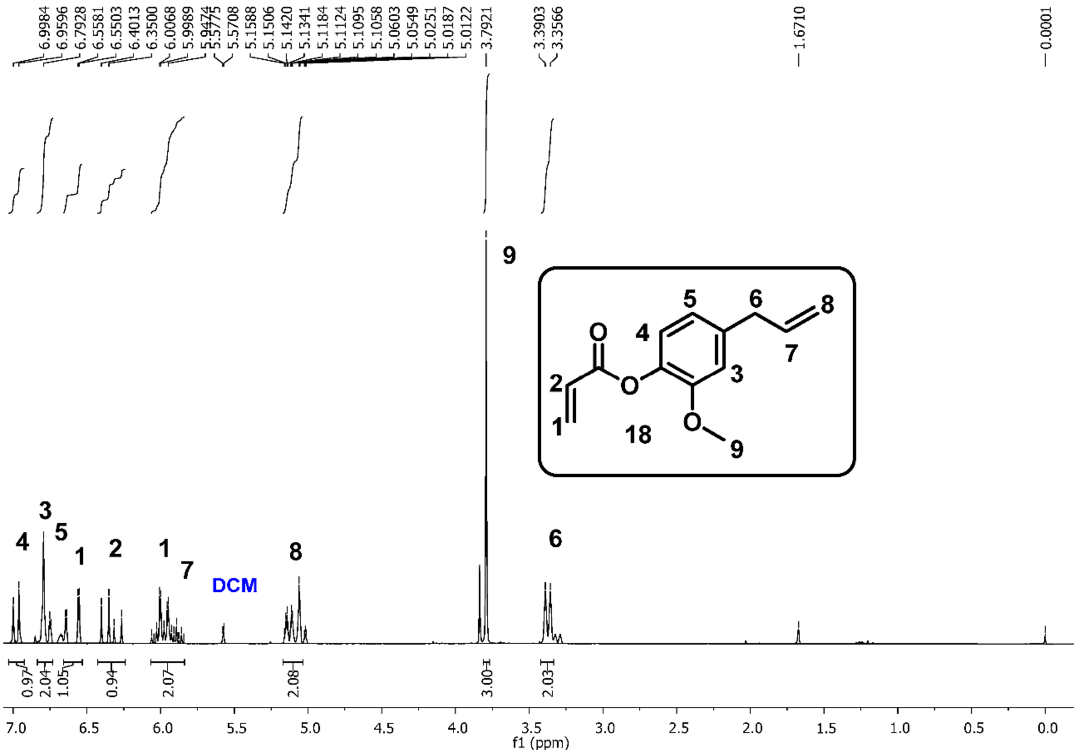

Figure S2. ^{1}H-NMR spectrum (200 MHz, $CDCl_3$) of 4-allyl-2-methoxyphenyl acrylate **18**.

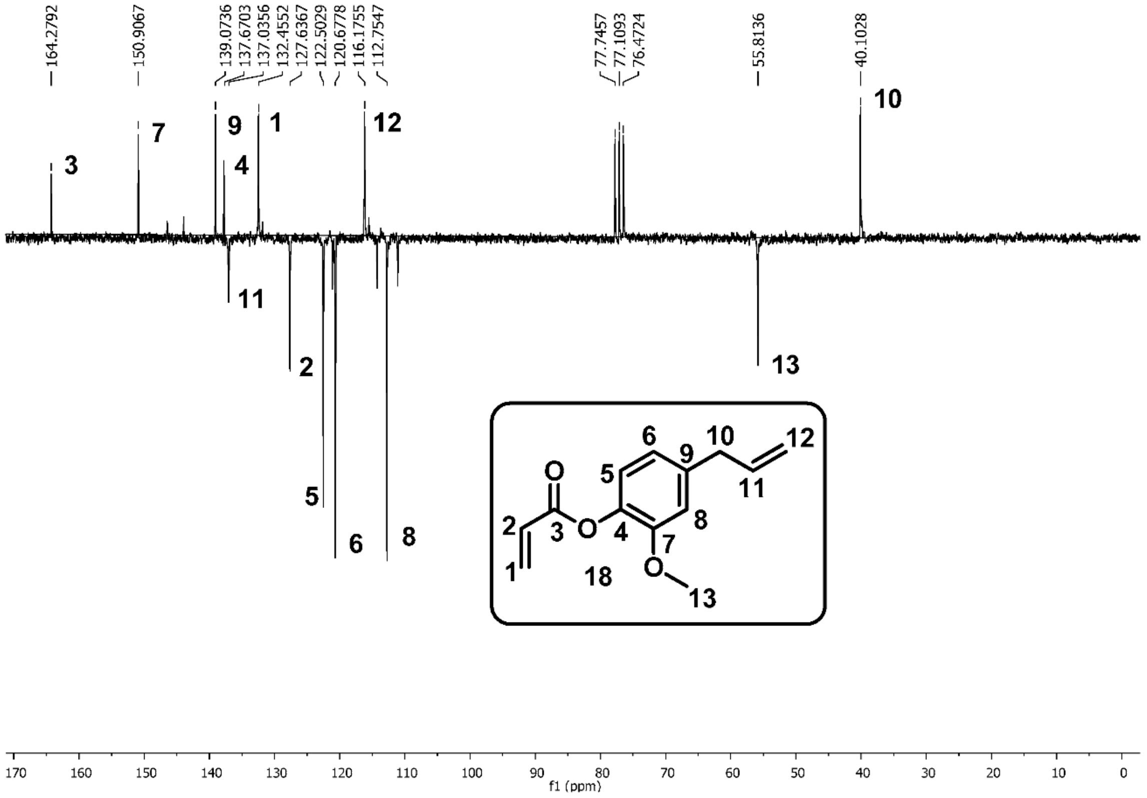

Figure S3. ^{13}C-NMR spectrum (50 MHz, $CDCl_3$) of 4-allyl-2-methoxyphenyl acrylate **18**.

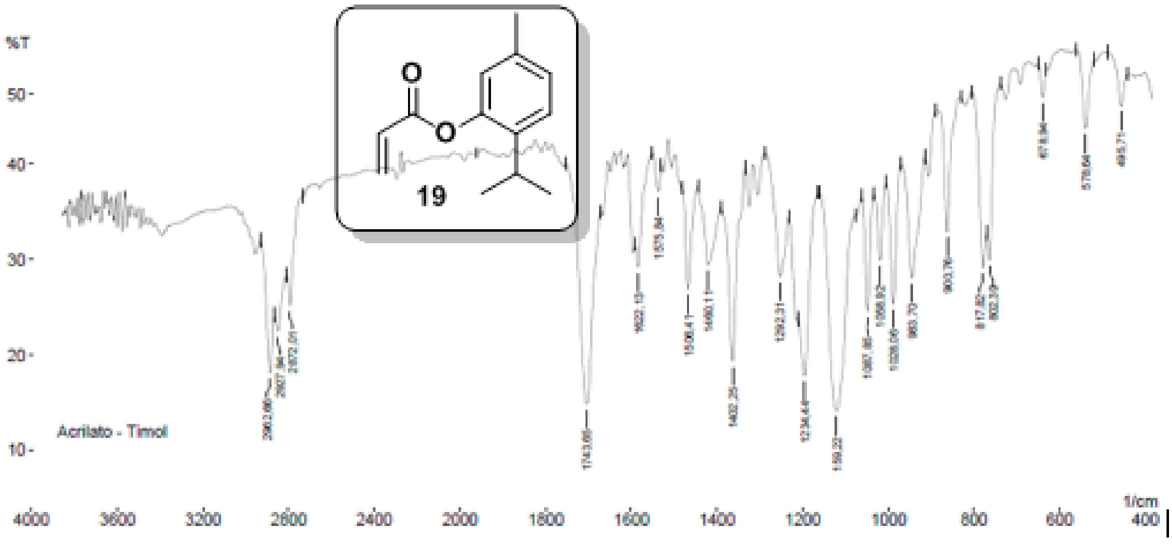

Figure S4. FTIR (KBr) spectrum of 2-isopropyl-5-methylphenyl acrylate **19**.

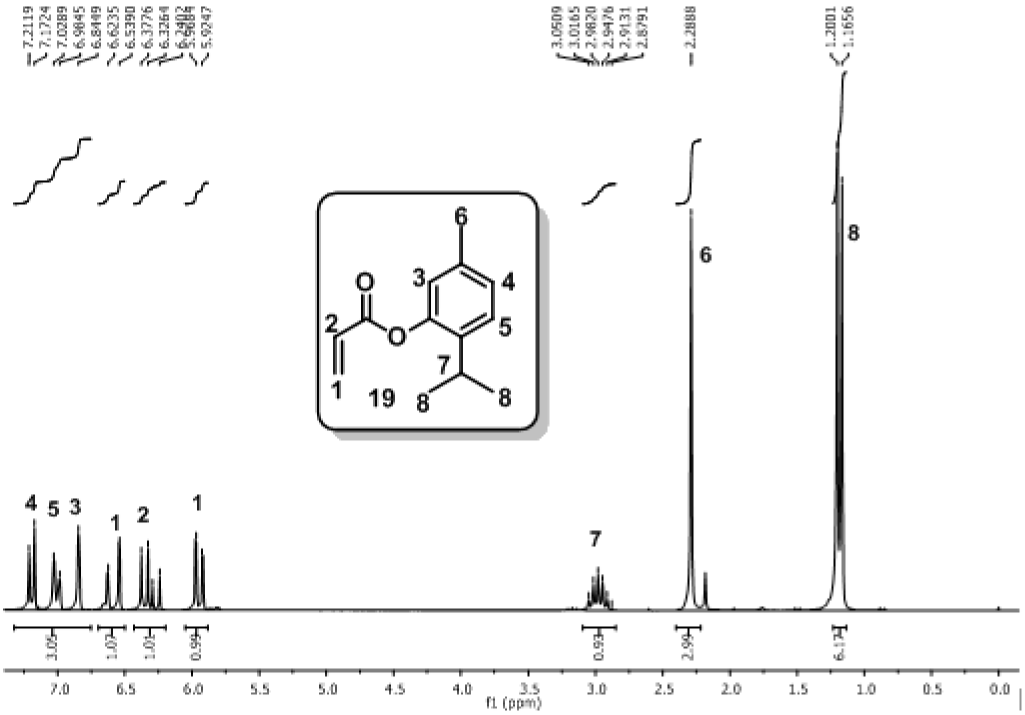

Figure S5. ^{1}H-NMR spectrum (200 MHz, $CDCl_3$) of 2-isopropyl-5-methylphenyl acrylate **19**.

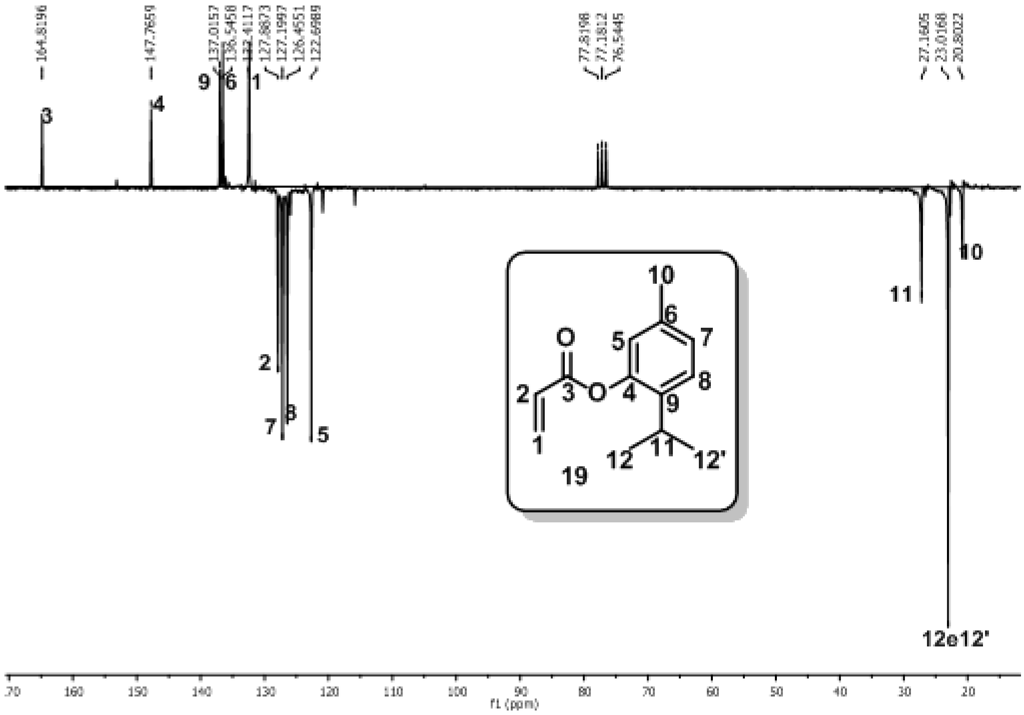

Figure S6. ^{13}C-NMR spectrum (50 MHz, $CDCl_3$) of 2-isopropyl-5-methylphenyl acrylate **19**.

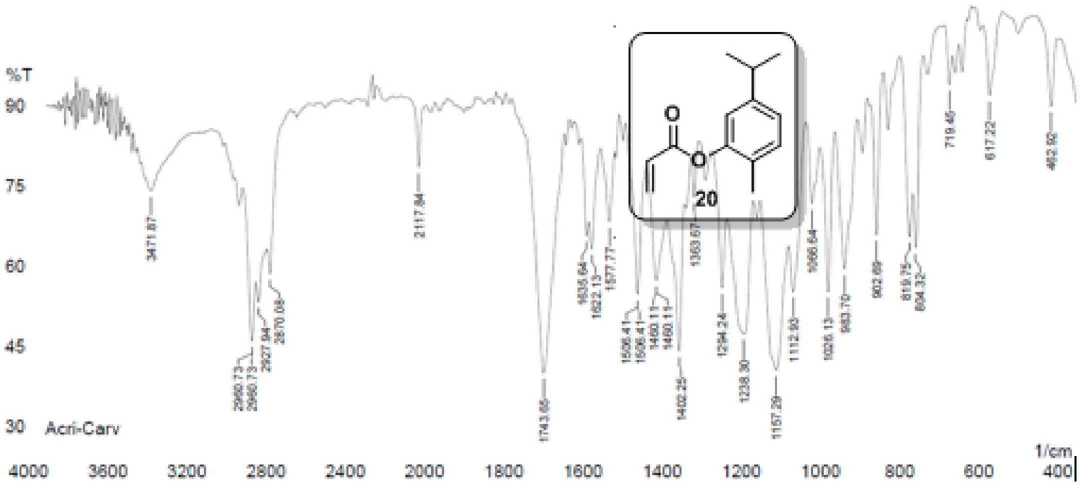

Figure S7. FTIR (KBr) spectrum of 5-isopropyl-2-methylphenyl acrylate **20**.

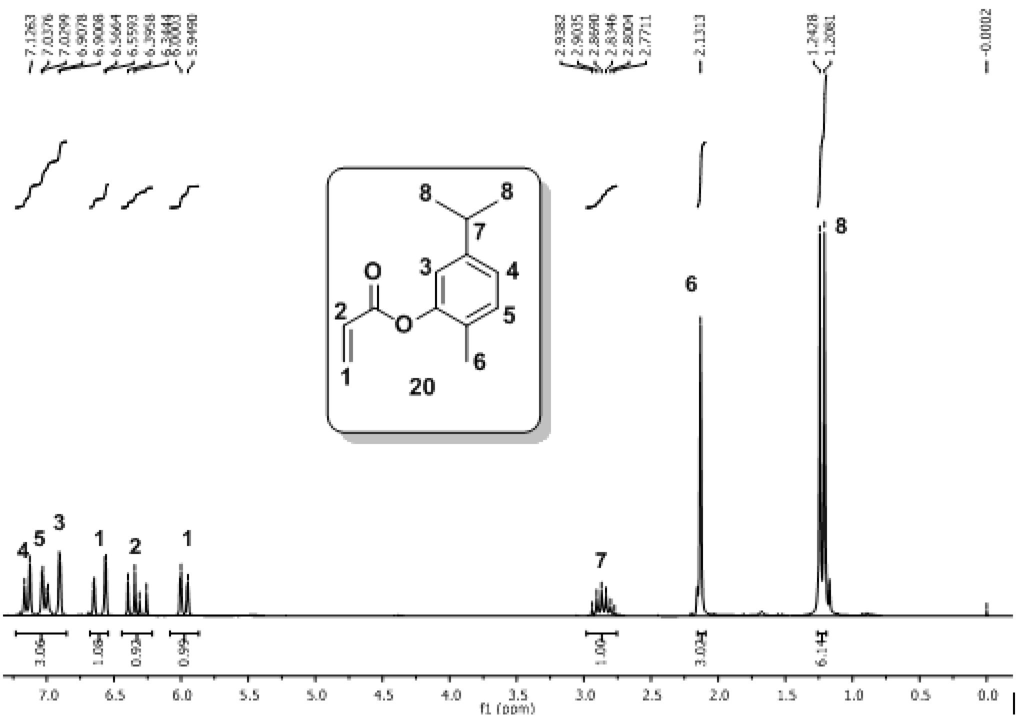

Figure S8. ^{1}H-NMR spectrum (200 MHz, $CDCl_3$) of 5-isopropyl-2-methylphenyl acrylate **20**.

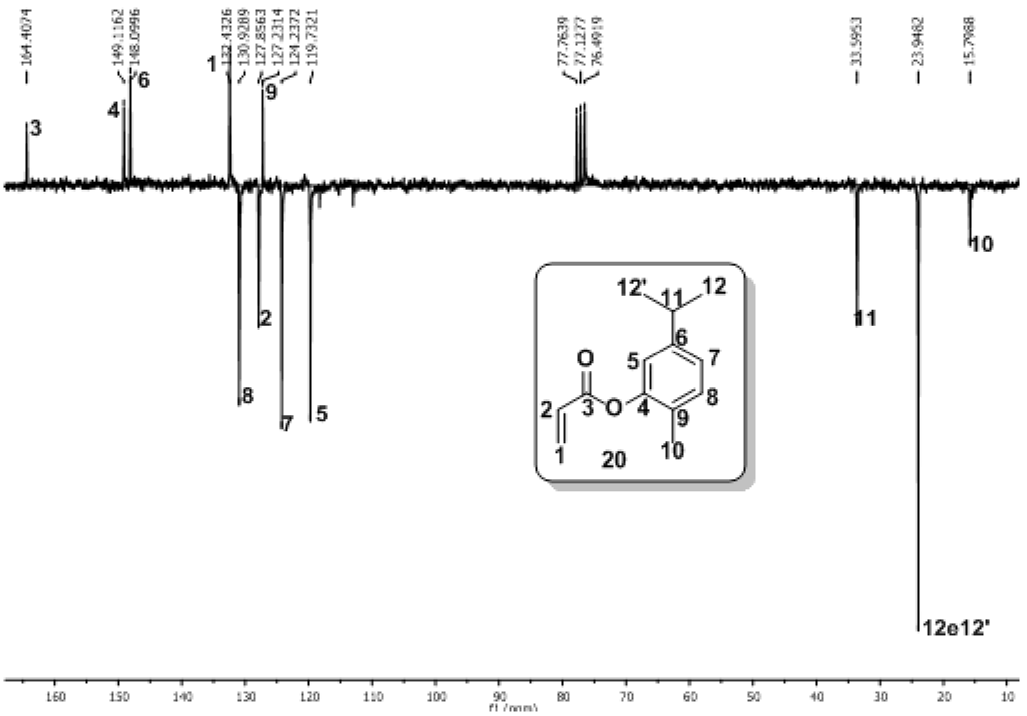

Figure S9. ^{13}C-NMR spectrum (50 MHz, $CDCl_3$) of 5-isopropyl-2-methylphenyl acrylate **20**.

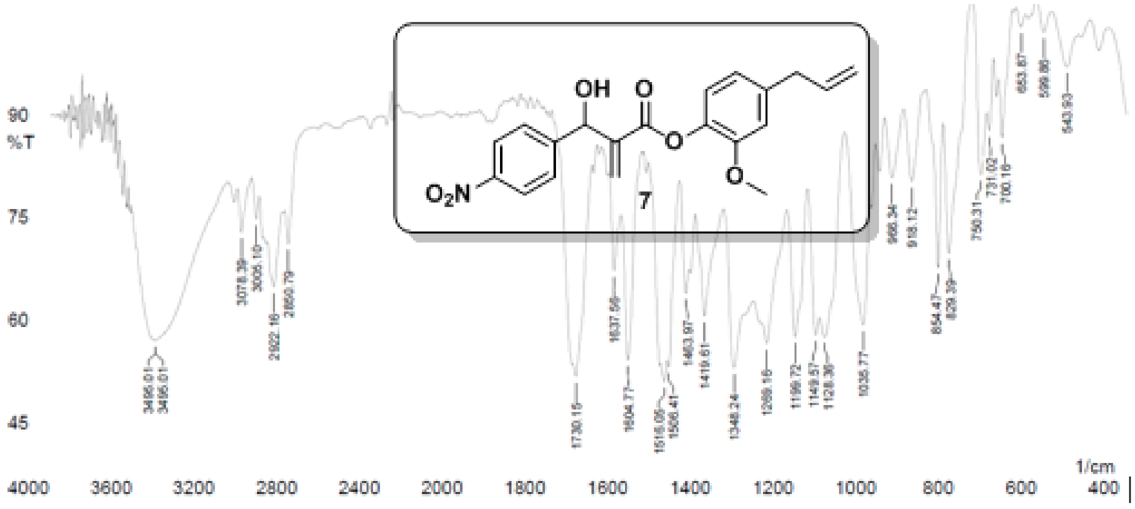

Figure S10. FTIR (KBr) spectrum of 4-allyl-2-methoxyphenyl 2-(hydroxy(4-nitrophenyl)methyl)acrylate **7**.

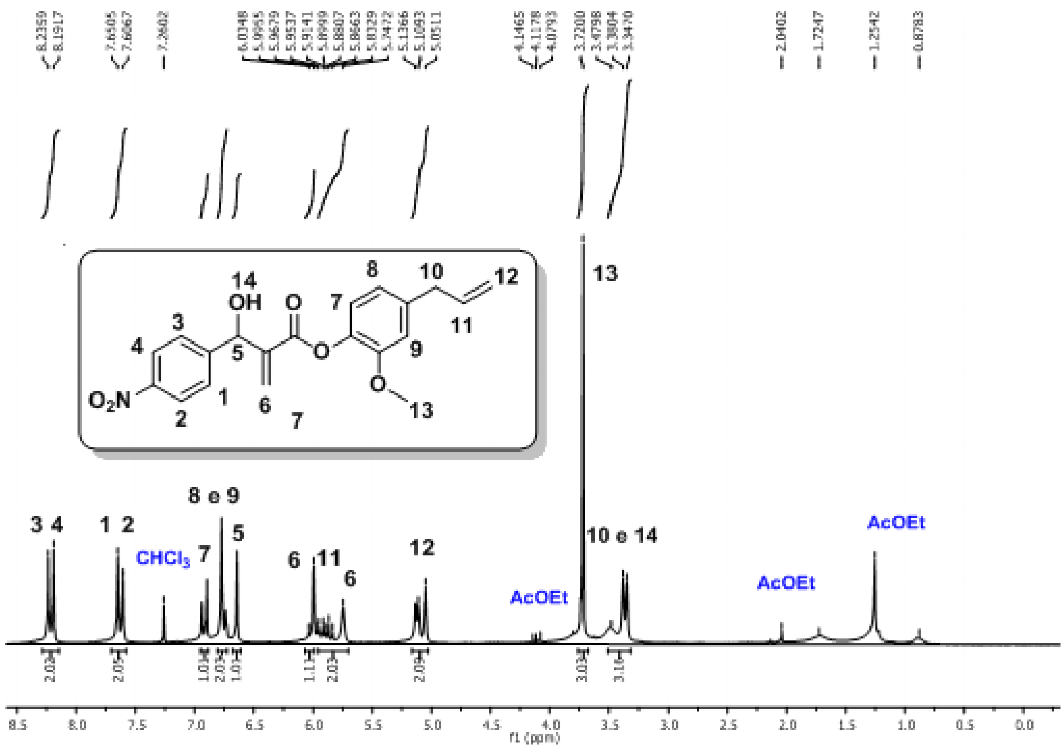

Figure S11. ¹H-NMR spectrum (200 MHz, $CDCl_3$) of 4-allyl-2-methoxyphenyl 2-(hydroxy(4-nitrophenyl)methyl)acrylate **7**.

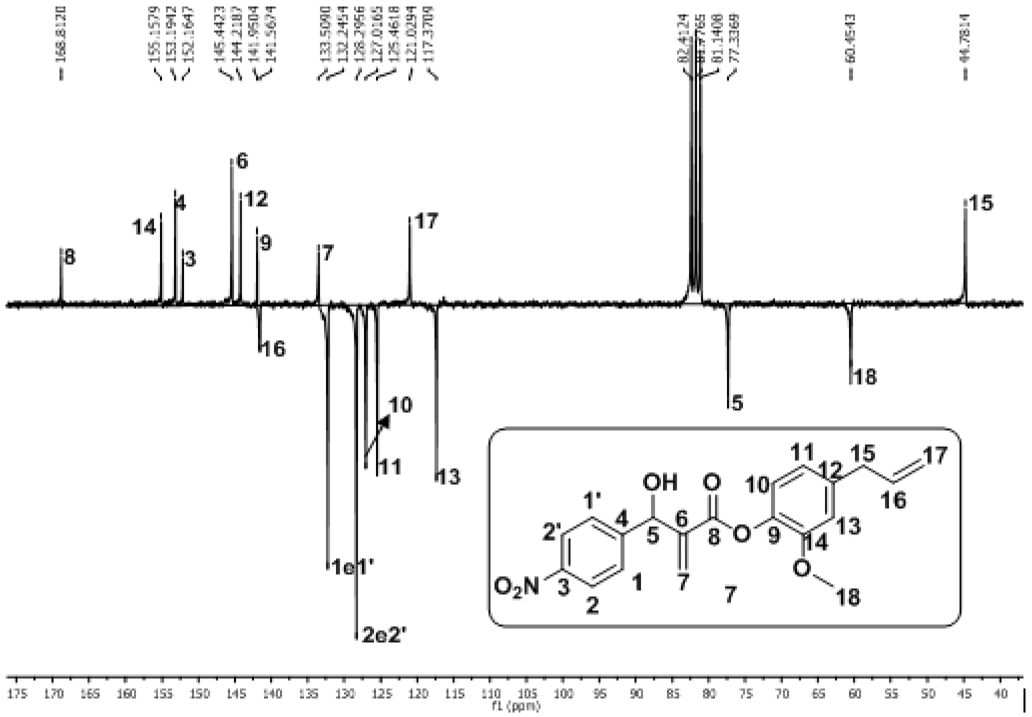

Figure S12. ¹³C-NMR spectrum (50 MHz, $CDCl_3$) of 4-allyl-2-methoxyphenyl 2-(hydroxy(4-nitrophenyl)methyl)acrylate **7**.

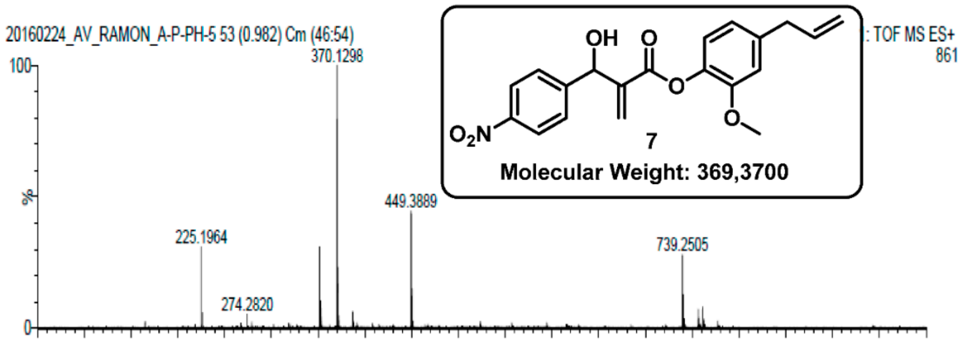

Figure S13. HRMS spectrum of compound 4-allyl-2-methoxyphenyl 2-(hydroxy(4-nitrophenyl)methyl)acrylate **7**.

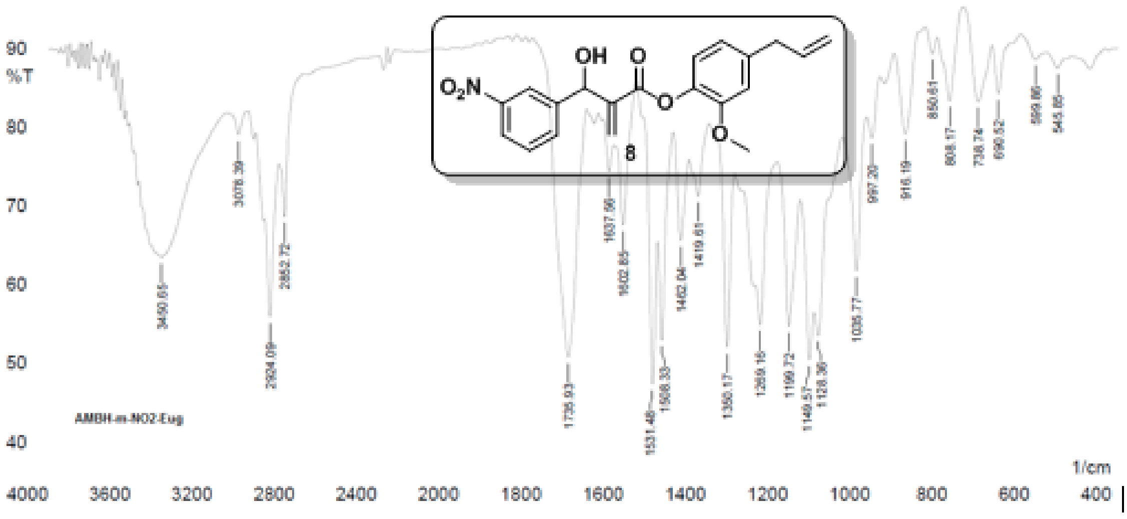

Figure S14. FTIR (KBr) spectrum of 4-allyl-2-methoxyphenyl 2-(hydroxy(3-nitrophenyl)methyl)acrylate **8**.

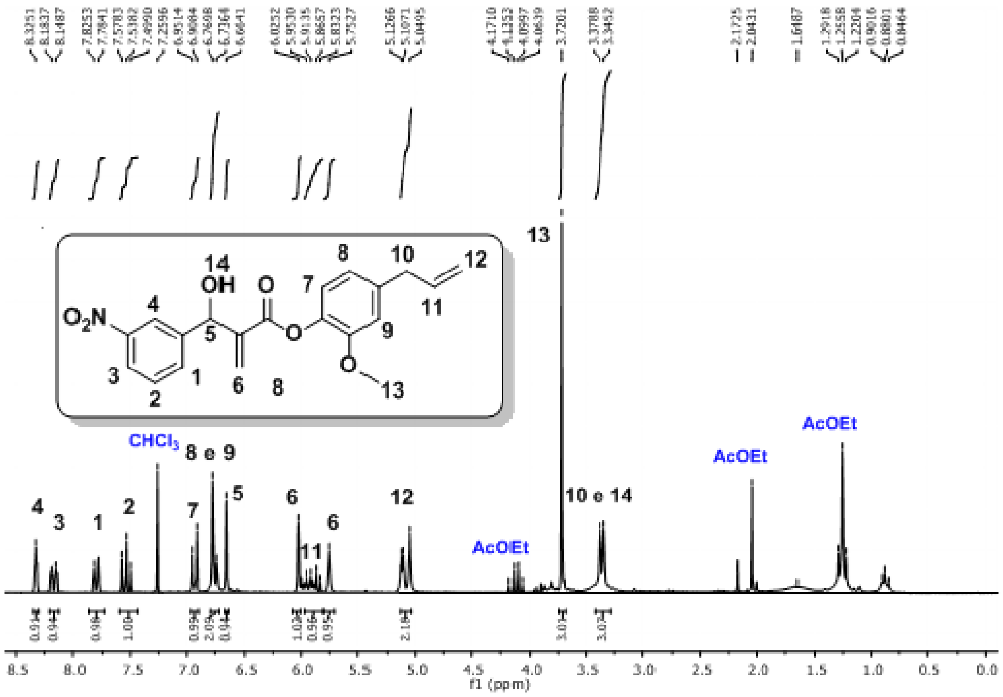

Figure S15. ^{1}H-NMR spectrum (200 MHz, $CDCl_3$) of 4-allyl-2-methoxyphenyl 2-(hydroxy(3-nitrophenyl)methyl)acrylate **8**.

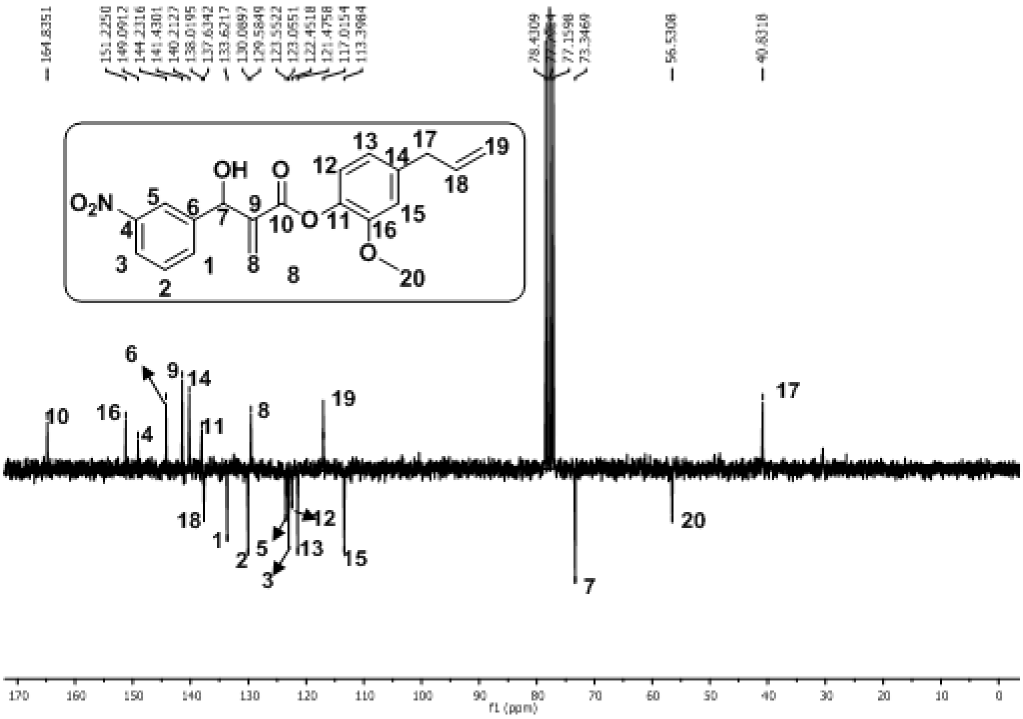

Figure S16. ^{13}C-NMR spectrum (50 MHz, $CDCl_3$) of 4-allyl-2-methoxyphenyl 2-(hydroxy(3-nitrophenyl)methyl)acrylate **8**.

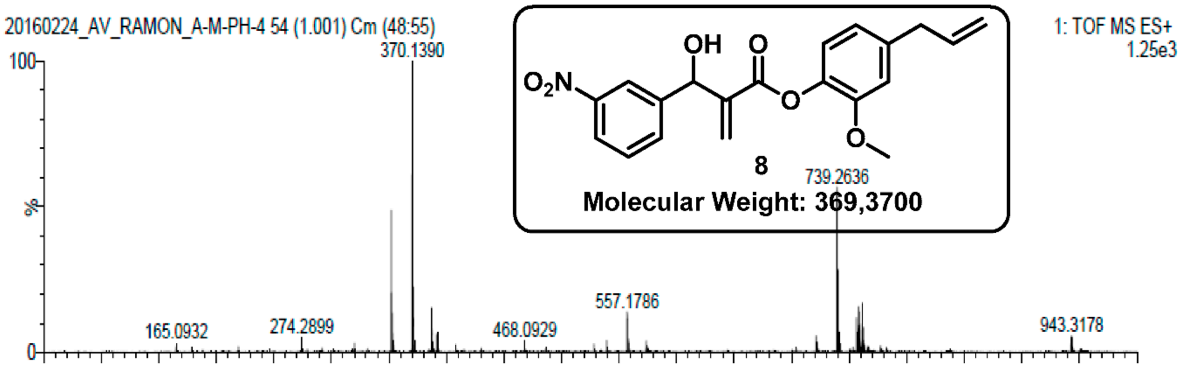

Figure S17. HRMS spectrum of compound 4-allyl-2-methoxyphenyl 2-(hydroxy(3-nitrophenyl)methyl)acrylate **8**.

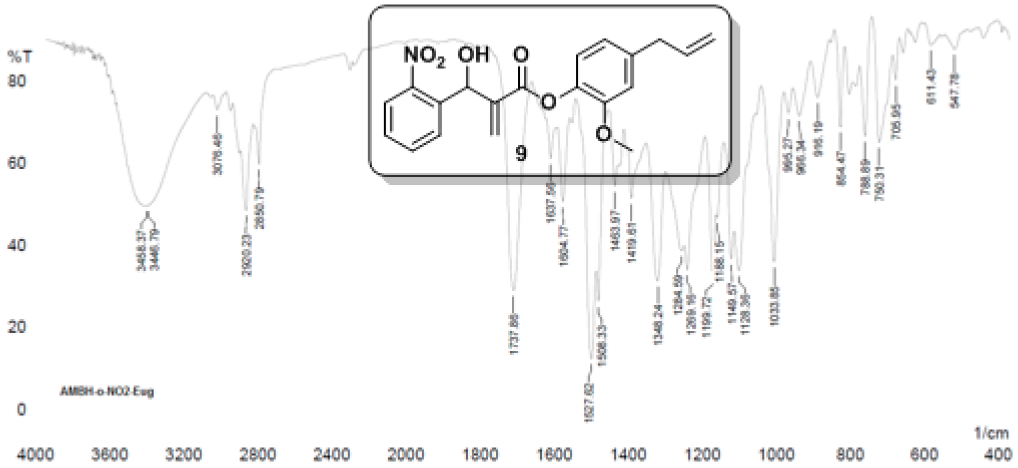

Figure S18. FTIR (KBr) spectrum of 4-allyl-2-methoxyphenyl 2-(hydroxy(2-nitrophenyl)methyl)acrylate **9**.

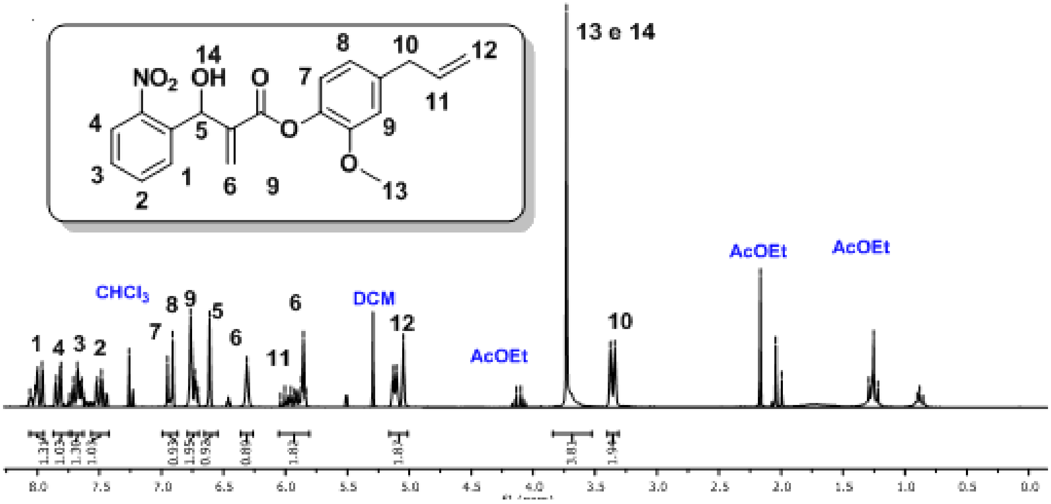

Figure S19. ^{1}H-NMR spectrum (200 MHz, $CDCl_3$) of 4-allyl-2-methoxyphenyl 2-(hydroxy(2-nitrophenyl)methyl)acrylate **9**.

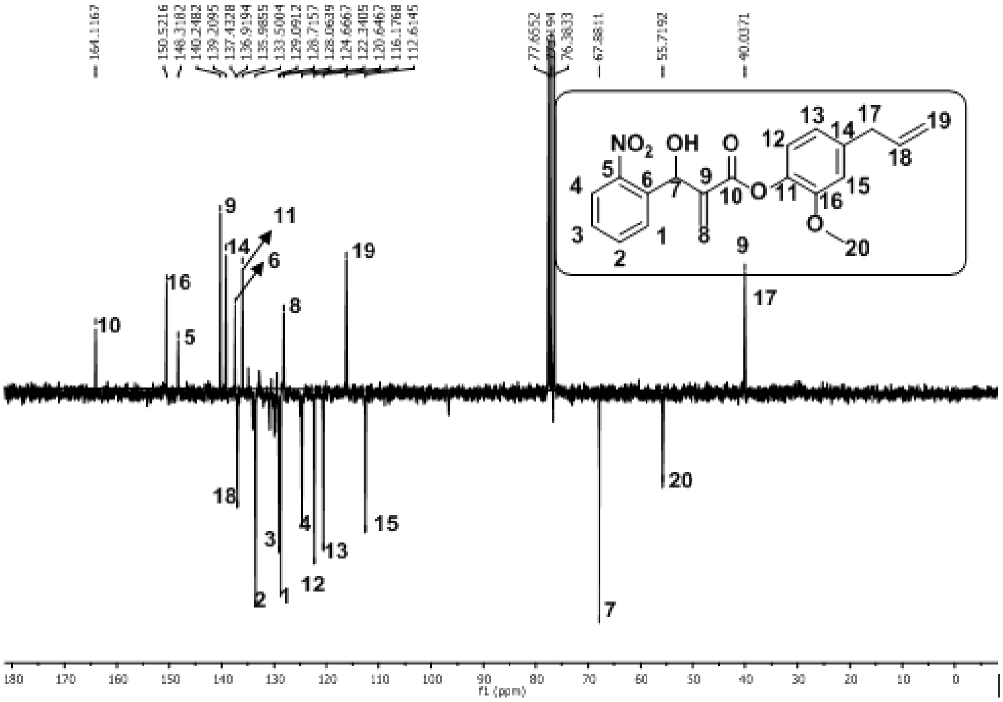

Figure S20. ^{13}C-NMR spectrum (50 MHz, $CDCl_3$) of 4-allyl-2-methoxyphenyl 2-(hydroxy(2-nitrophenyl)methyl)acrylate **9**.

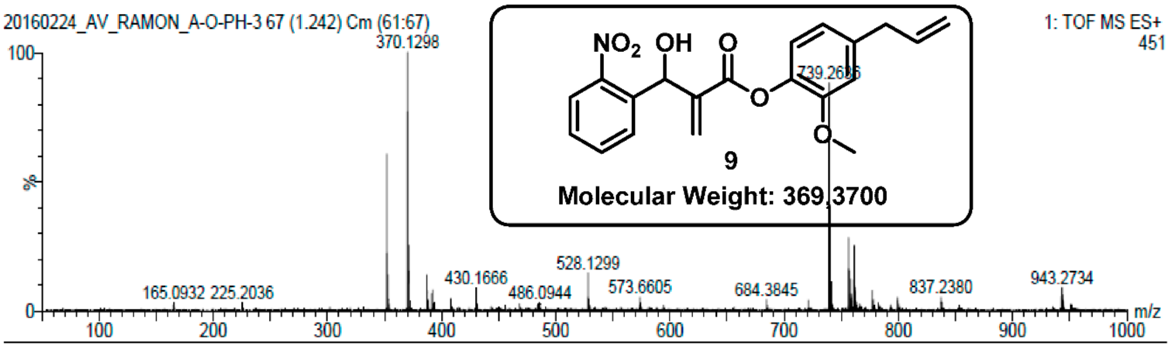

Figure S21. HRMS spectrum of compound 4-allyl-2-methoxyphenyl 2-(hydroxy(2-nitrophenyl)methyl)acrylate **9**.

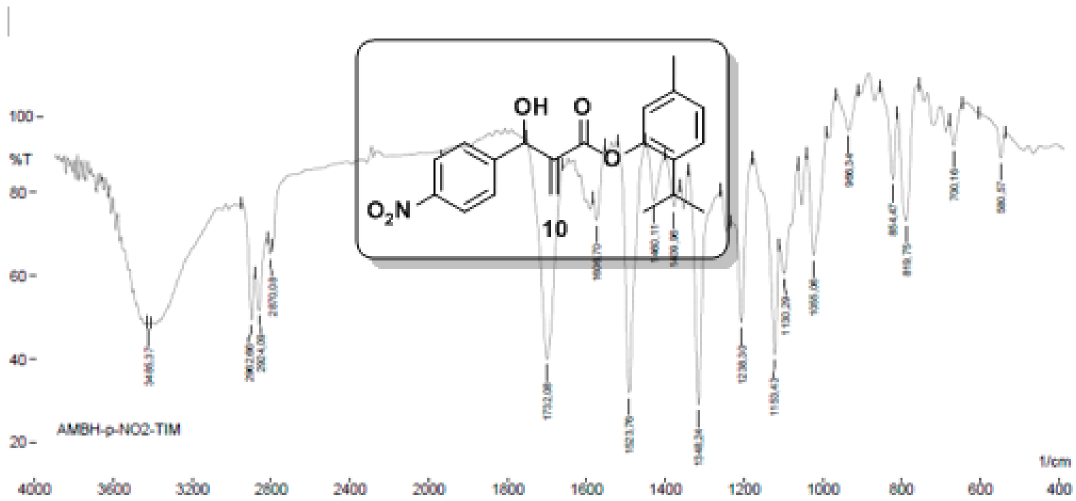

Figure S22. FTIR (KBr) spectrum of 2-isopropyl-5-methylphenyl 2-(hydroxy(4-nitrophenyl)methyl)acrylate **10**.

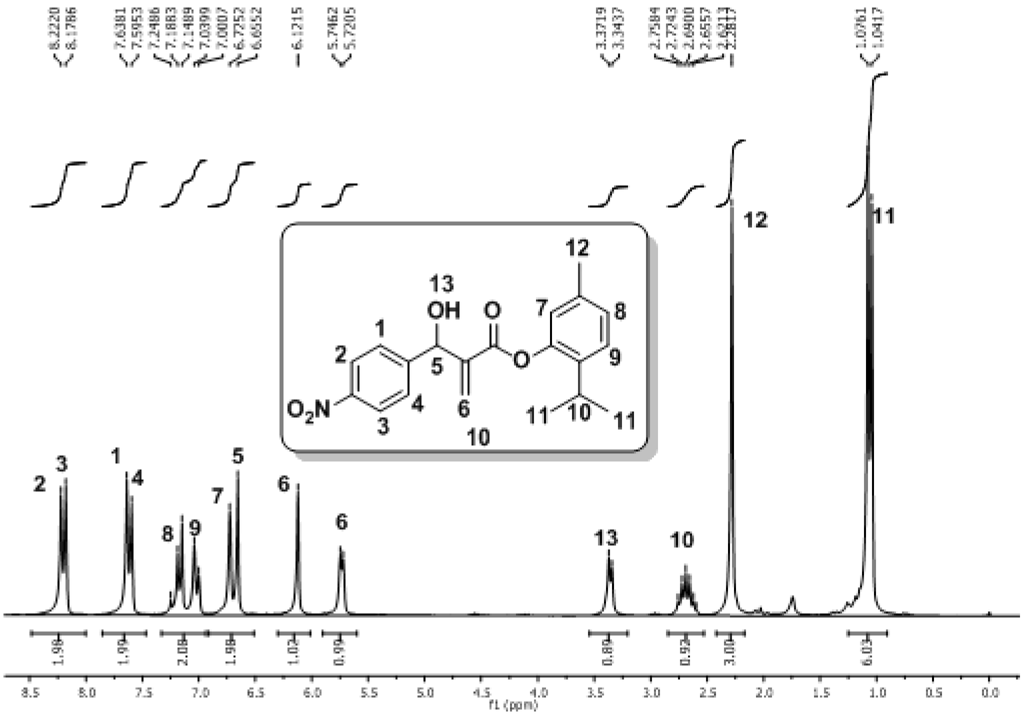

Figure S23. ^{1}H-NMR spectrum (200 MHz, $CDCl_3$) of 2-(hydroxy(4-nitrophenyl)methyl)acrylate **10**.

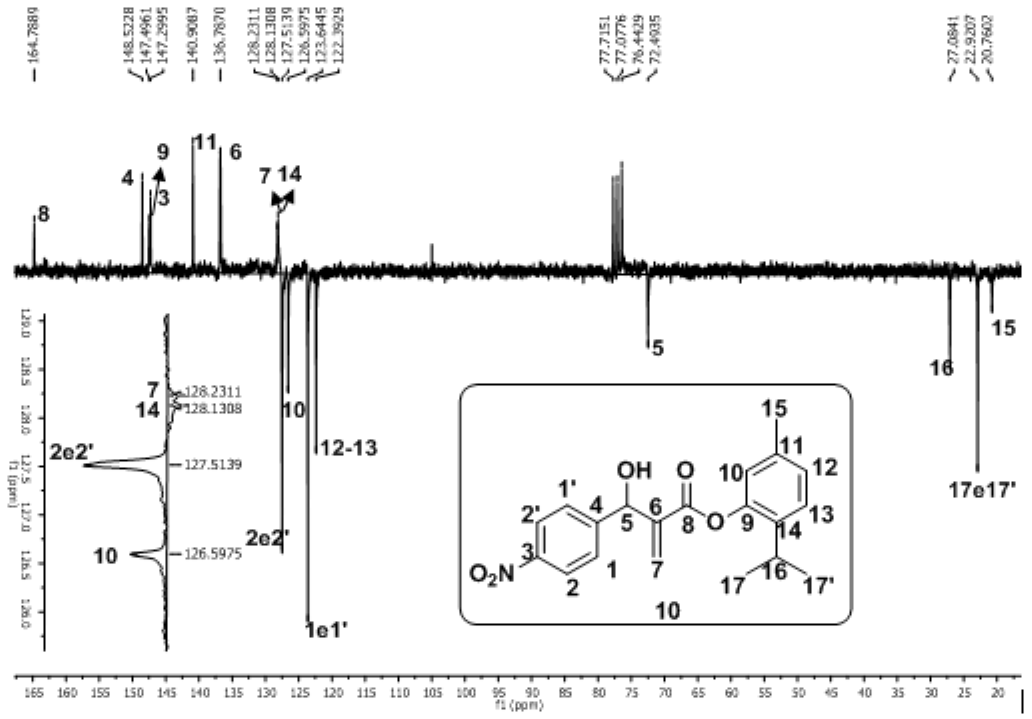

Figure S24. ^{13}C-NMR spectrum (50 MHz, $CDCl_3$) of 2-(hydroxy(4-nitrophenyl)methyl)acrylate **10**.

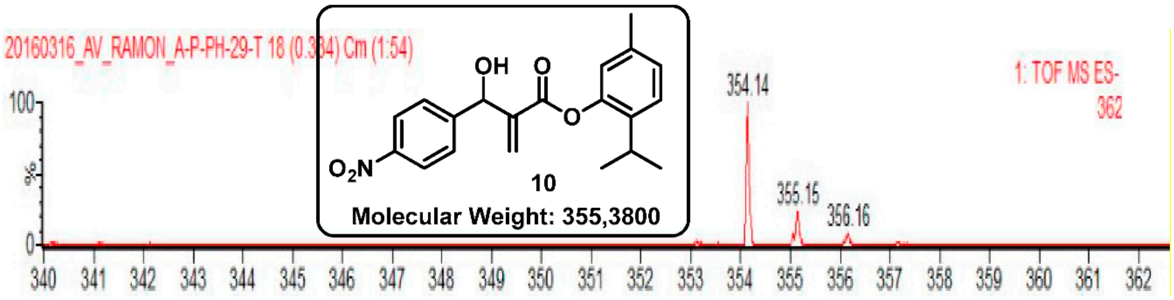

Figure S25. HRMS spectrum of compound 2-(hydroxy(4-nitrophenyl)methyl)acrylate **10**.

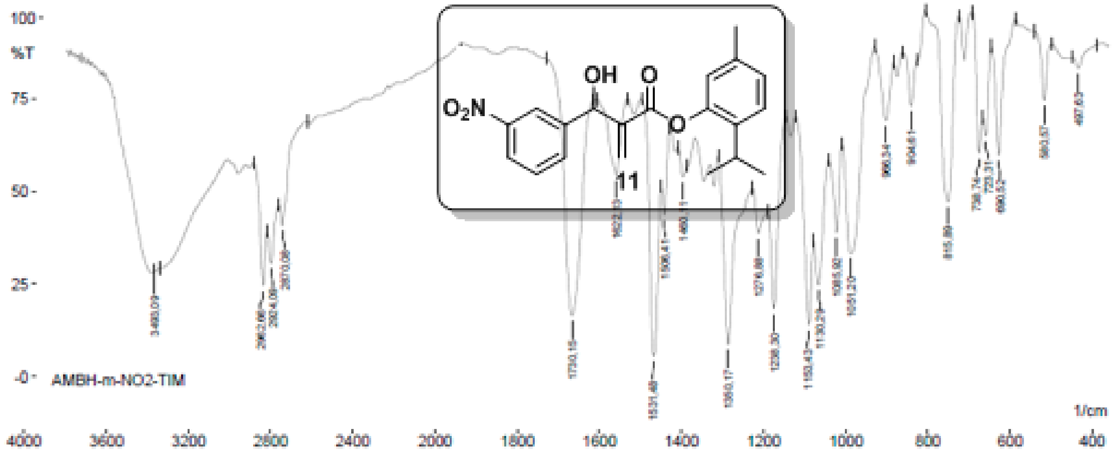

Figure S26. FTIR (KBr) spectrum of 2-isopropyl-5-methylphenyl 2-(hydroxy(3-nitrophenyl)methyl)acrylate **11**.

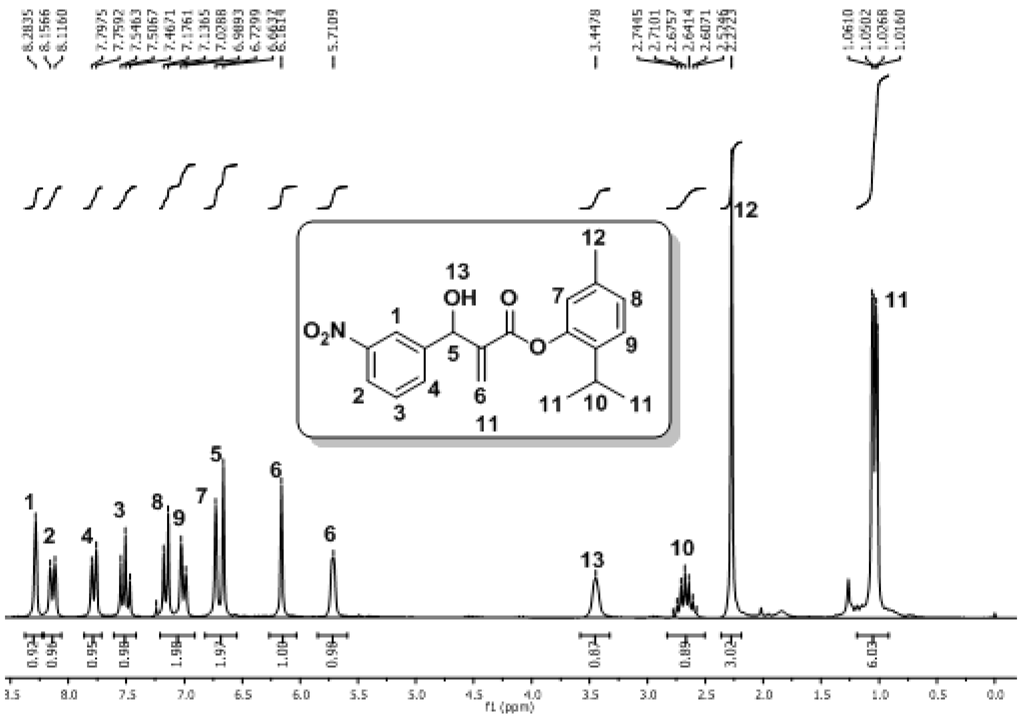

Figure S27. ^{1}H-NMR spectrum (200 MHz, $CDCl_3$) of 2-(hydroxy(3-nitrophenyl)methyl)acrylate **11**.

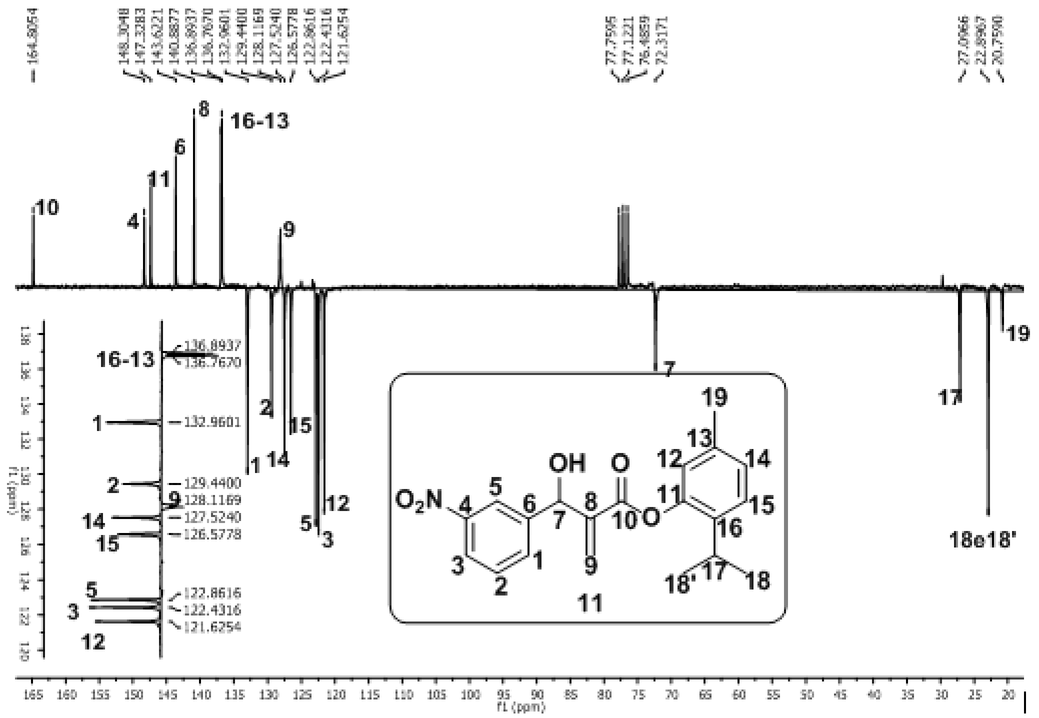

Figure S28. ^{13}C-NMR spectrum (50 MHz, $CDCl_3$) of 2-(hydroxy(3-nitrophenyl)methyl)acrylate **11**.

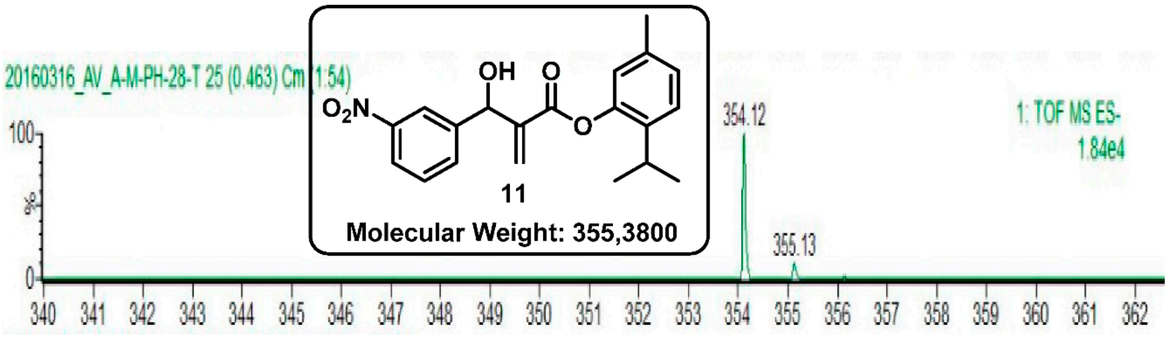

Figure S29. HRMS spectrum of compound 2-(hydroxy(3-nitrophenyl)methyl)acrylate **11**.

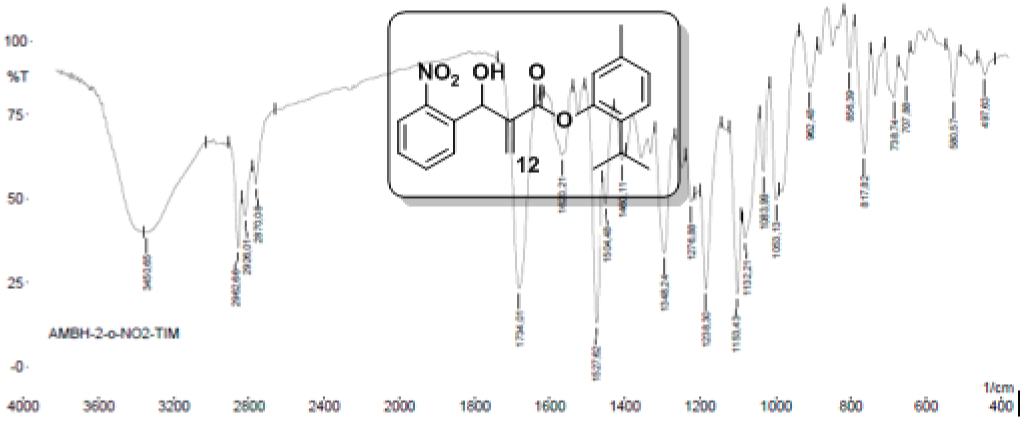

Figure S30. FTIR (KBr) spectrum of 2-isopropyl-5-methylphenyl 2-(hydroxy(2-nitrophenyl)methyl)acrylate **12**.

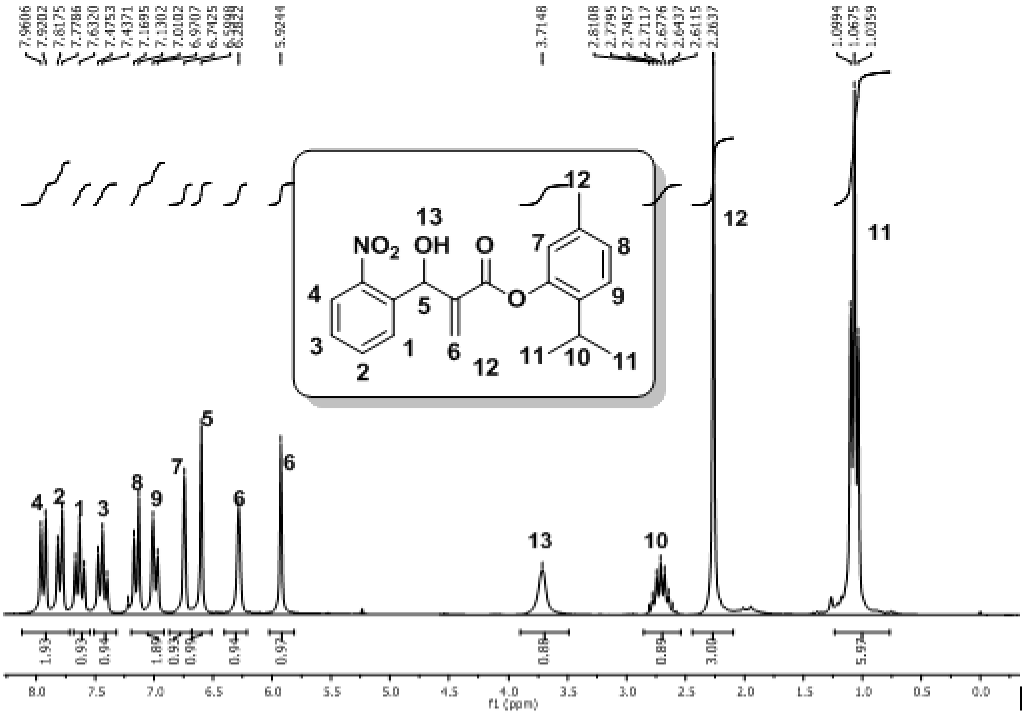

Figure S31. ^{1}H-NMR spectrum (200 MHz, $CDCl_3$) of 2-(hydroxy(2-nitrophenyl)methyl)acrylate **12**.

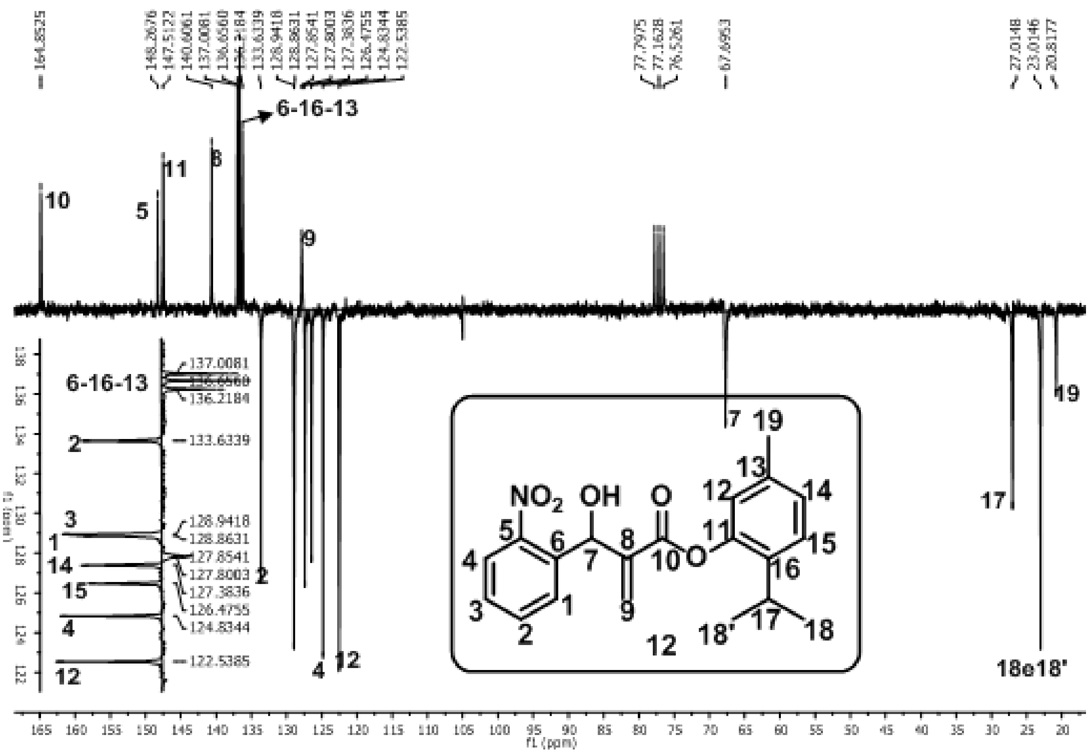

Figure S32. ^{13}C-NMR spectrum (50 MHz, $CDCl_3$) of 2-(hydroxy(2-nitrophenyl)methyl)acrylate **12**.

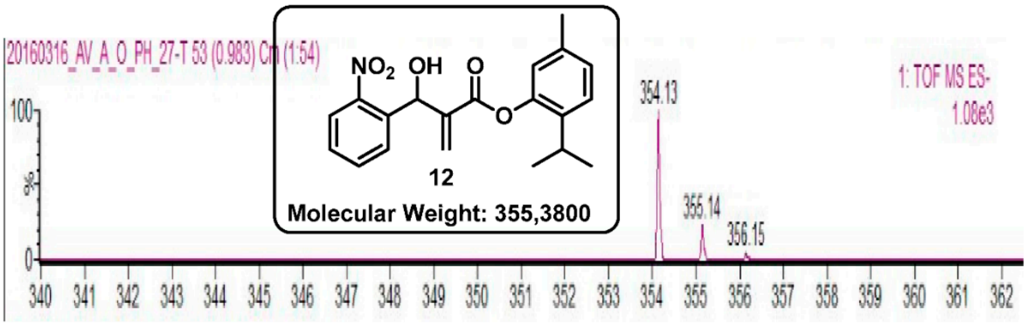

Figure S33. HRMS spectrum of compound 2-(hydroxy(2-nitrophenyl)methyl)acrylate **12**.

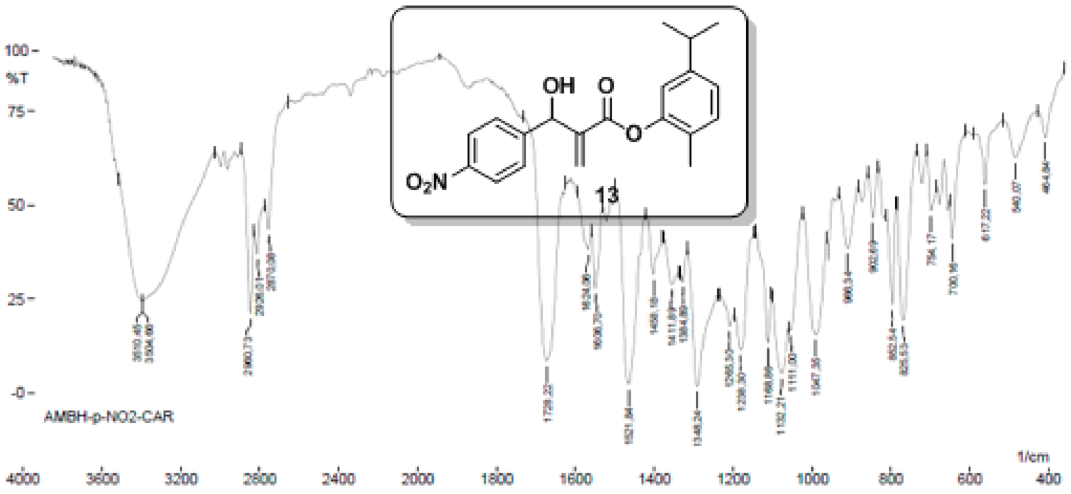

Figure S34. FTIR (KBr) spectrum of 5-isopropyl-2-methylphenyl 2-(hydroxy(4-nitrophenyl)methyl)acrylate **13**.

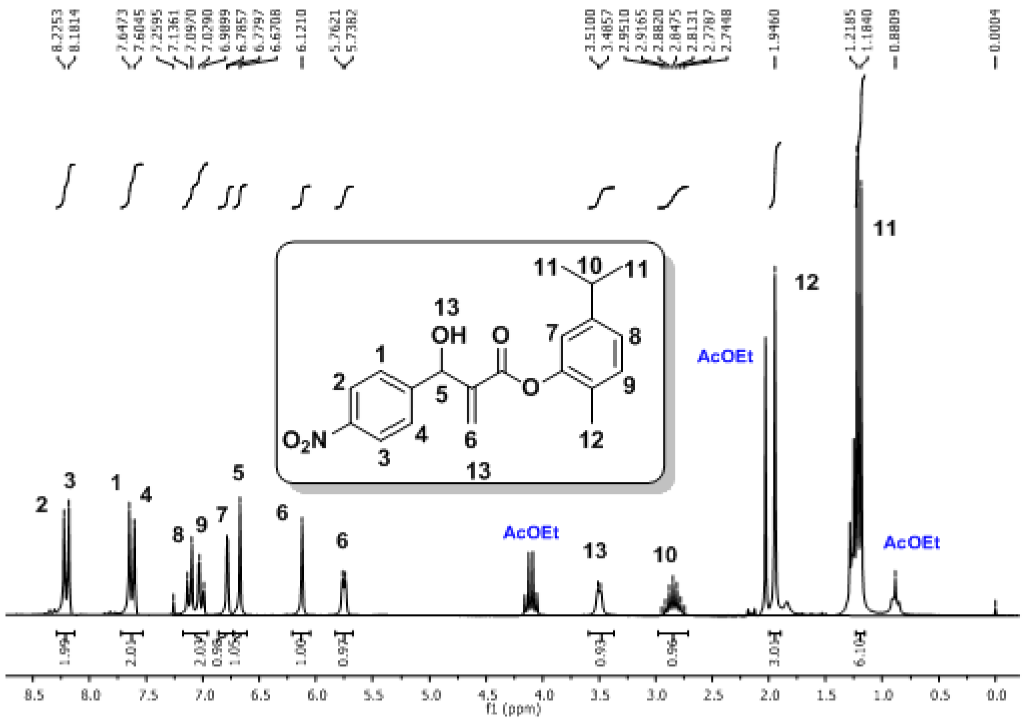

Figure S35. ^{1}H-NMR spectrum (200 MHz, $CDCl_3$) of 5-isopropyl-2-methylphenyl 2-(hydroxy(4-nitrophenyl)methyl)acrylate **13**.

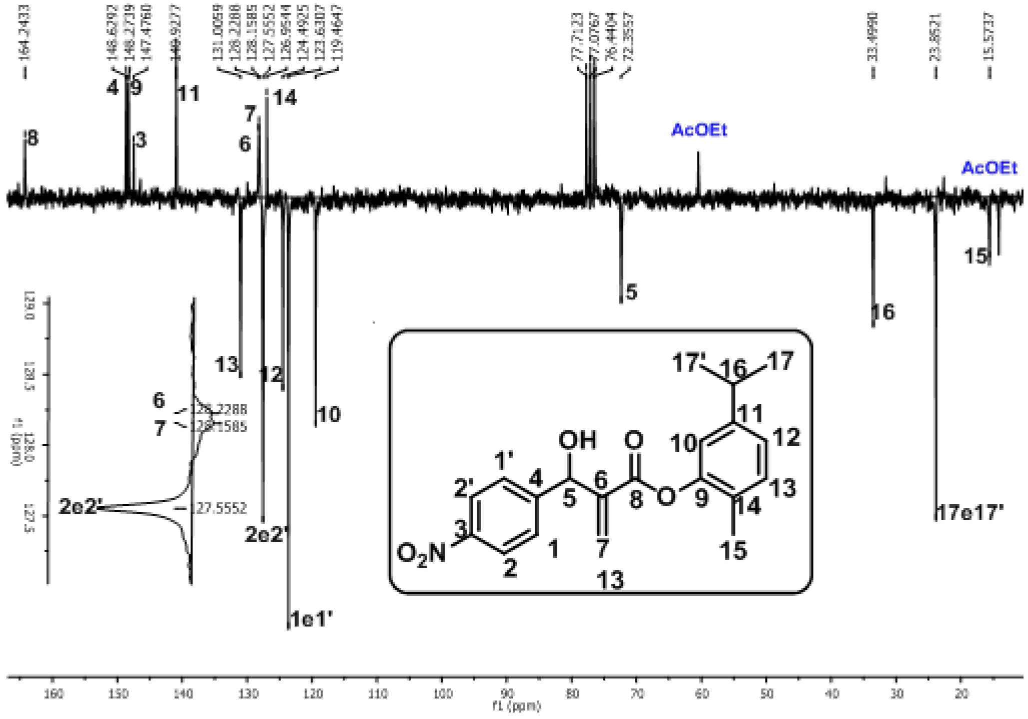

Figure S36. ^{13}C-NMR spectrum (50 MHz, $CDCl_3$) of of 5-isopropyl-2-methylphenyl 2-(hydroxy(4-nitrophenyl)methyl)acrylate **13**.

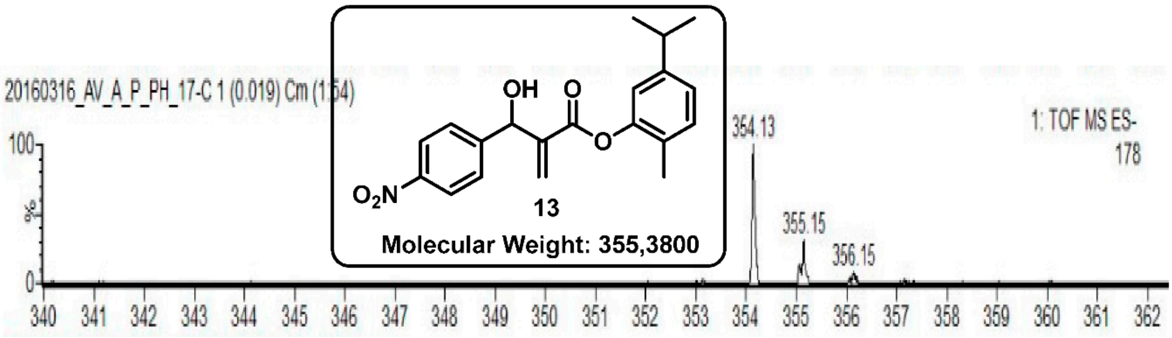

Figure S37. HRMS spectrum of of 5-isopropyl-2-methylphenyl 2-(hydroxy(4-nitrophenyl)methyl)acrylate **13**.

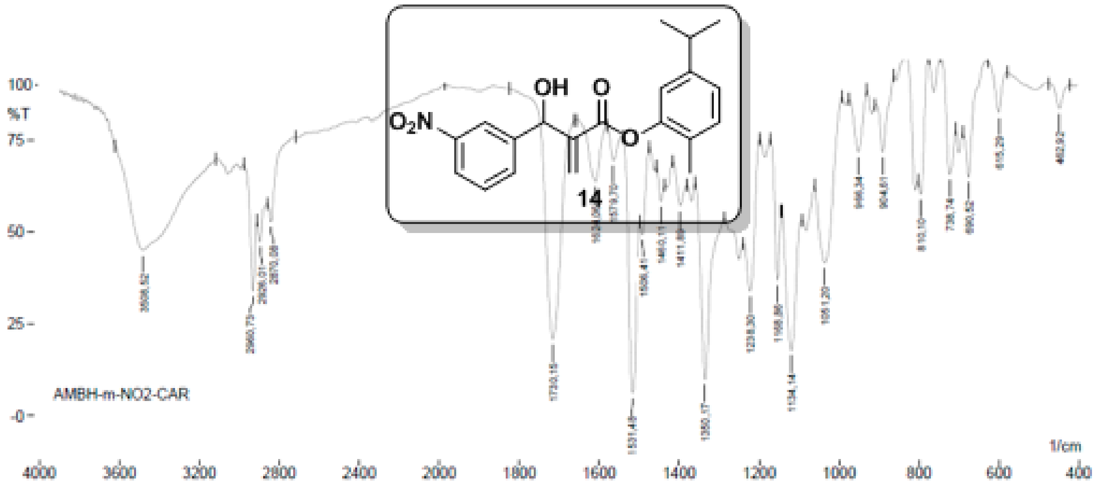

Figure S38. FTIR (KBr) spectrum of 5-isopropyl-2-methylphenyl 2-(hydroxy(3-nitrophenyl)methyl)acrylate **14**.

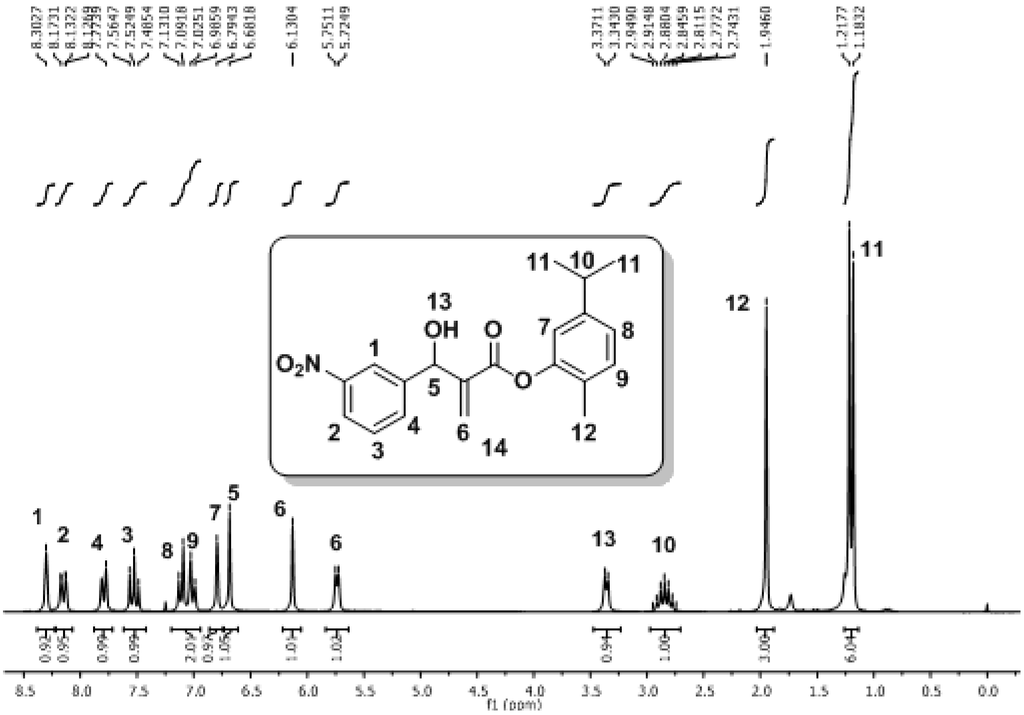

Figure S39. ^{1}H-NMR spectrum (200 MHz, $CDCl_3$) of 5-isopropyl-2-methylphenyl 2-(hydroxy(3-nitrophenyl)methyl)acrylate **14**.

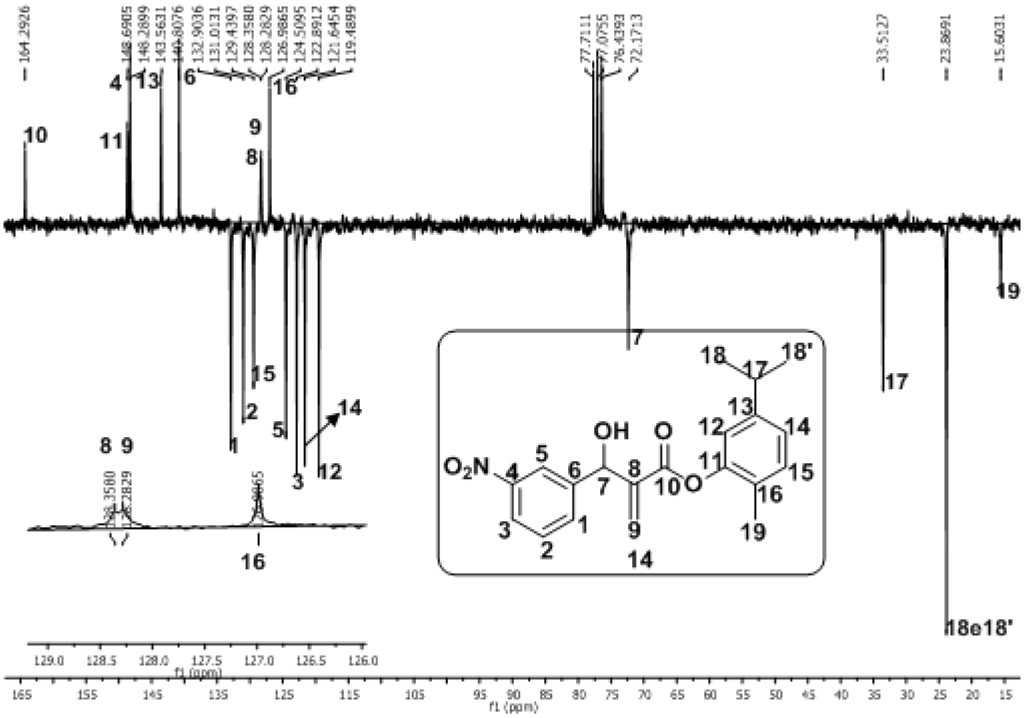

Figure S40. ^{13}C-NMR spectrum (50 MHz, $CDCl_3$) of 5-isopropyl-2-methylphenyl 2-(hydroxy(3-nitrophenyl)methyl)acrylate **14**.

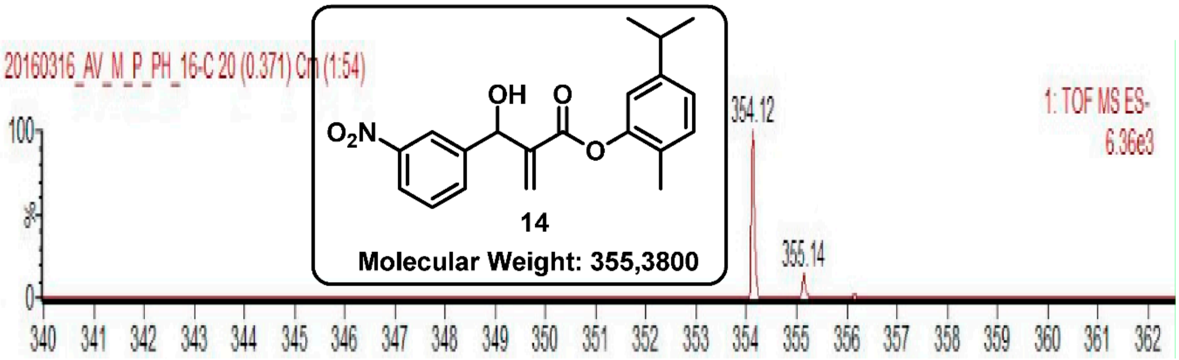

Figure S41. HRMS spectrum of compound 5-isopropyl-2-methylphenyl 2-(hydroxy(3-nitrophenyl)methyl)acrylate **14**.

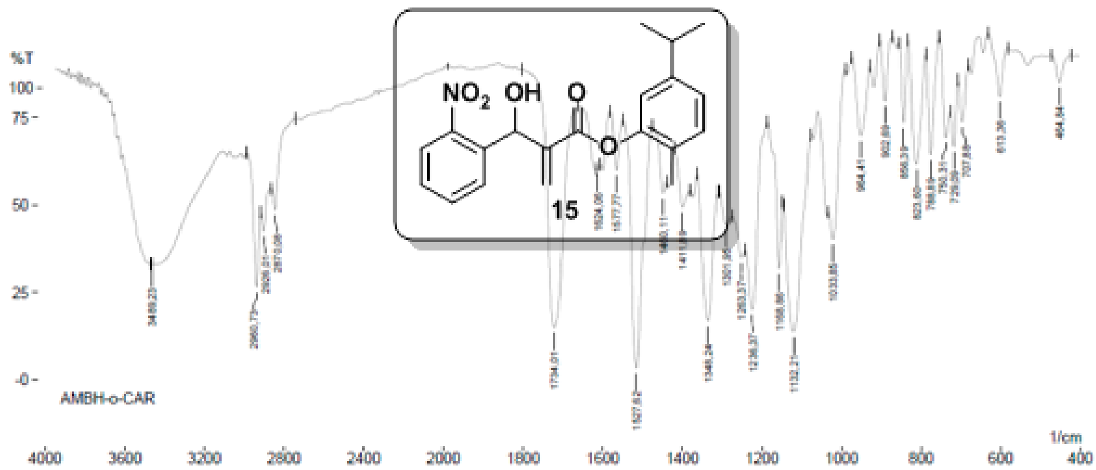

Figure S42. FTIR (KBr) spectrum of 5-isopropyl-2-methylphenyl 2-(hydroxy(2-nitrophenyl)methyl)acrylate **15**.

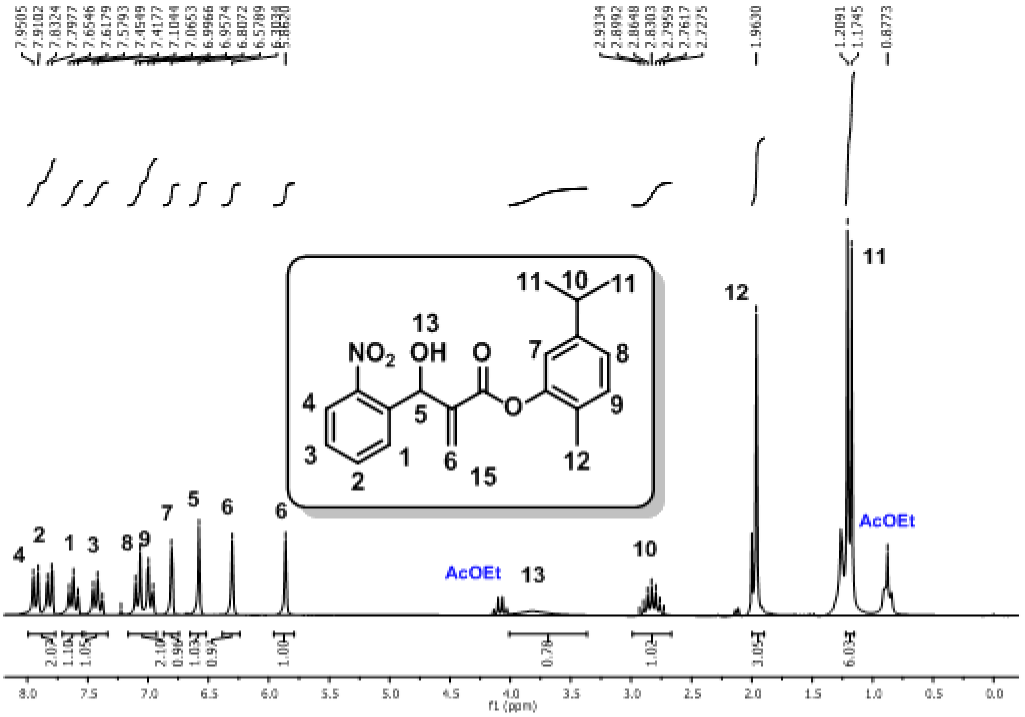

Figure S43. ^{1}H-NMR spectrum (200 MHz, $CDCl_3$) of 5-isopropyl-2-methylphenyl 2-(hydroxy(2-nitrophenyl)methyl)acrylate **15**.

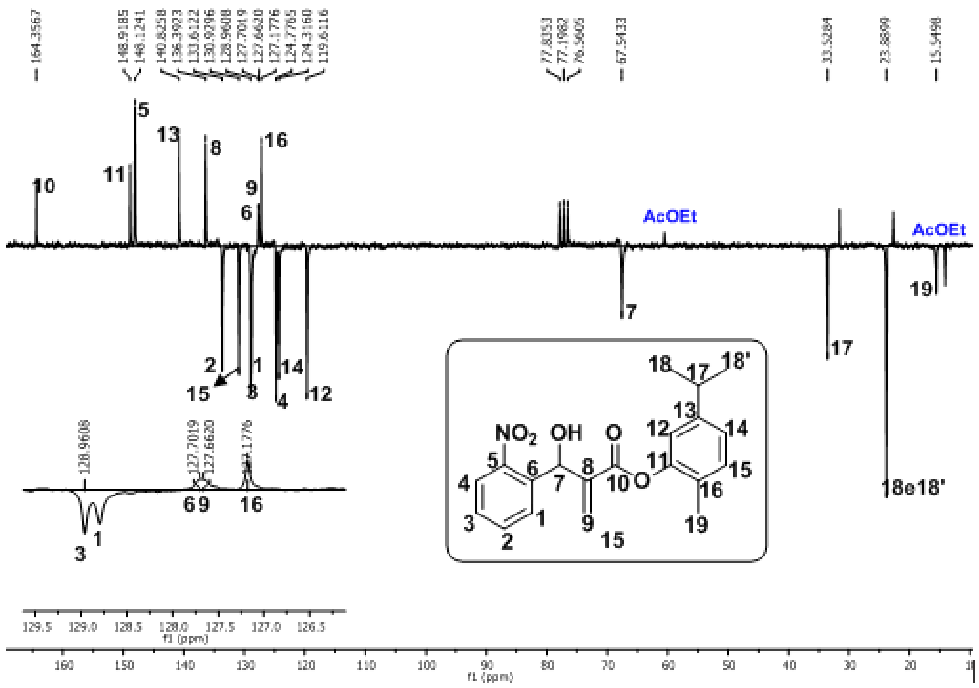

Figure S44. ^{13}C-NMR spectrum (50 MHz, $CDCl_3$) of 5-isopropyl-2-methylphenyl 2-(hydroxy(2-nitrophenyl)methyl)acrylate **15**.

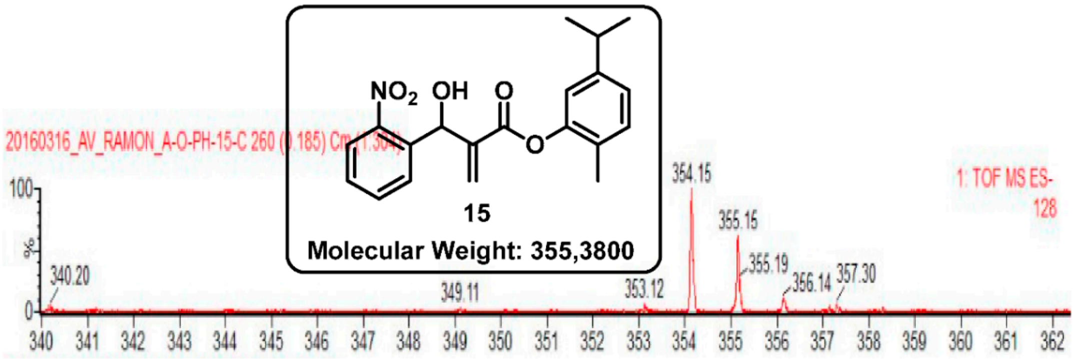

Figure S45. HRMS spectrum of compound 5-isopropyl-2-methylphenyl 2-(hydroxy(2-nitrophenyl)methyl)acrylate **15**.

m062x_water_*para* (7)

Created by GaussView 5.0.8

```
 46 47  0  0  0  0  0  0  0  0  0     0
  -5.8250   -0.5297   -0.0750 C   0  0  0  0  0  0  0  0  0  0  0  0
  -5.0075   -1.5307    0.4415 C   0  0  0  0  0  0  0  0  0  0  0  0
  -3.7112   -1.1912    0.8042 C   0  0  0  0  0  0  0  0  0  0  0  0
  -3.2462    0.1184    0.6470 C   0  0  0  0  0  0  0  0  0  0  0  0
  -4.0910    1.0996    0.1227 C   0  0  0  0  0  0  0  0  0  0  0  0
  -5.3952    0.7815   -0.2416 C   0  0  0  0  0  0  0  0  0  0  0  0
  -5.3784   -2.5418    0.5620 H   0  0  0  0  0  0  0  0  0  0  0  0
  -3.0515   -1.9509    1.2150 H   0  0  0  0  0  0  0  0  0  0  0  0
  -3.7327    2.1157   -0.0035 H   0  0  0  0  0  0  0  0  0  0  0  0
  -6.0615    1.5331   -0.6487 H   0  0  0  0  0  0  0  0  0  0  0  0
  -1.8167    0.4324    1.0144 C   0  0  0  0  0  0  0  0  0  0  0  0
  -1.5459   -0.1765    1.8867 H   0  0  0  0  0  0  0  0  0  0  0  0
```

-0.8445	0.0994	-0.1100	C	0	0	0	0	0	0	0	0	0	0	0	0	0
-1.1788	-0.2719	-1.3482	C	0	0	0	0	0	0	0	0	0	0	0	0	0
-2.2181	-0.3861	-1.6441	H	0	0	0	0	0	0	0	0	0	0	0	0	0
-0.4234	-0.4725	-2.1012	H	0	0	0	0	0	0	0	0	0	0	0	0	0
0.5784	0.2540	0.2928	C	0	0	0	0	0	0	0	0	0	0	0	0	0
0.9193	0.6752	1.3815	O	0	0	0	0	0	0	0	0	0	0	0	0	0
1.4607	-0.1340	-0.6513	O	0	0	0	0	0	0	0	0	0	0	0	0	0
-1.7192	1.8115	1.3571	O	0	0	0	0	0	0	0	0	0	0	0	0	0
-0.8355	1.9498	1.7395	H	0	0	0	0	0	0	0	0	0	0	0	0	0
2.8179	0.0247	-0.3566	C	0	0	0	0	0	0	0	0	0	0	0	0	0
3.3629	1.3131	-0.3493	C	0	0	0	0	0	0	0	0	0	0	0	0	0
3.5917	-1.1008	-0.1401	C	0	0	0	0	0	0	0	0	0	0	0	0	0
4.7299	1.4466	-0.1057	C	0	0	0	0	0	0	0	0	0	0	0	0	0
4.9633	-0.9730	0.1039	C	0	0	0	0	0	0	0	0	0	0	0	0	0
3.1131	-2.0768	-0.1610	H	0	0	0	0	0	0	0	0	0	0	0	0	0
5.5113	0.3106	0.1145	C	0	0	0	0	0	0	0	0	0	0	0	0	0
5.1919	2.4274	-0.0891	H	0	0	0	0	0	0	0	0	0	0	0	0	0
6.5756	0.4373	0.2999	H	0	0	0	0	0	0	0	0	0	0	0	0	0
-7.1953	-0.8719	-0.4544	N	0	0	0	0	0	0	0	0	0	0	0	0	0
-7.9229	0.0165	-0.8747	O	0	0	0	0	0	0	0	0	0	0	0	0	0
-7.5581	-2.0336	-0.3359	O	0	0	0	0	0	0	0	0	0	0	0	0	0
3.0317	3.6587	-0.5415	C	0	0	0	0	0	0	0	0	0	0	0	0	0
3.4599	3.8698	0.4435	H	0	0	0	0	0	0	0	0	0	0	0	0	0
3.7905	3.7955	-1.3184	H	0	0	0	0	0	0	0	0	0	0	0	0	0
2.1890	4.3233	-0.7294	H	0	0	0	0	0	0	0	0	0	0	0	0	0
2.4988	2.3368	-0.5841	O	0	0	0	0	0	0	0	0	0	0	0	0	0
5.8235	-2.1934	0.3701	C	0	0	0	0	0	0	0	0	0	0	0	0	0
5.2224	-3.0910	0.1810	H	0	0	0	0	0	0	0	0	0	0	0	0	0
6.1173	-2.2165	1.4272	H	0	0	0	0	0	0	0	0	0	0	0	0	0
7.0611	-2.2252	-0.4843	C	0	0	0	0	0	0	0	0	0	0	0	0	0
6.8980	-2.2481	-1.5629	H	0	0	0	0	0	0	0	0	0	0	0	0	0
8.3051	-2.2141	-0.0031	C	0	0	0	0	0	0	0	0	0	0	0	0	0
8.4951	-2.1843	1.0687	H	0	0	0	0	0	0	0	0	0	0	0	0	0
9.1704	-2.2366	-0.6604	H	0	0	0	0	0	0	0	0	0	0	0	0	0

1	2	4	0	0	0	0
1	6	4	0	0	0	0
1	31	1	0	0	0	0
2	3	4	0	0	0	0
2	7	1	0	0	0	0
3	4	4	0	0	0	0
3	8	1	0	0	0	0
4	5	4	0	0	0	0
4	11	1	0	0	0	0
5	6	4	0	0	0	0
5	9	1	0	0	0	0
6	10	1	0	0	0	0
11	12	1	0	0	0	0
11	13	1	0	0	0	0
11	20	1	0	0	0	0
13	14	2	0	0	0	0
13	17	1	0	0	0	0
14	15	1	0	0	0	0

14	16	1	0	0	0	0
17	18	2	0	0	0	0
17	19	1	0	0	0	0
19	22	1	0	0	0	0
20	21	1	0	0	0	0
22	23	4	0	0	0	0
22	24	2	0	0	0	0
23	25	4	0	0	0	0
23	38	1	0	0	0	0
24	26	4	0	0	0	0
24	27	1	0	0	0	0
25	28	4	0	0	0	0
25	29	1	0	0	0	0
26	28	4	0	0	0	0
26	39	1	0	0	0	0
28	30	1	0	0	0	0
31	32	2	0	0	0	0
31	33	2	0	0	0	0
34	35	1	0	0	0	0
34	36	1	0	0	0	0
34	37	1	0	0	0	0
34	38	1	0	0	0	0
39	40	1	0	0	0	0
39	41	1	0	0	0	0
39	42	1	0	0	0	0
42	43	1	0	0	0	0
42	44	2	0	0	0	0
44	45	1	0	0	0	0
44	46	1	0	0	0	0

m062x_water_*ortho* (9)

Created by GaussView 5.0.8

46 47 0 0 0 0 0 0 0 0 0 0

3.2201	-1.9313	-2.0484	C	0	0	0	0	0	0	0	0	0	0	0	0
2.8688	-0.6415	-1.6533	C	0	0	0	0	0	0	0	0	0	0	0	0
3.1298	-0.1723	-0.3644	C	0	0	0	0	0	0	0	0	0	0	0	0
3.7708	-1.0703	0.5000	C	0	0	0	0	0	0	0	0	0	0	0	0
4.1075	-2.3723	0.1412	C	0	0	0	0	0	0	0	0	0	0	0	0
3.8325	-2.8033	-1.1504	C	0	0	0	0	0	0	0	0	0	0	0	0
3.0055	-2.2563	-3.0618	H	0	0	0	0	0	0	0	0	0	0	0	0
2.3711	0.0142	-2.3625	H	0	0	0	0	0	0	0	0	0	0	0	0
4.5974	-3.0203	0.8599	H	0	0	0	0	0	0	0	0	0	0	0	0
4.1020	-3.8103	-1.4511	H	0	0	0	0	0	0	0	0	0	0	0	0
2.7151	1.2432	0.0448	C	0	0	0	0	0	0	0	0	0	0	0	0
2.4302	1.2387	1.1030	H	0	0	0	0	0	0	0	0	0	0	0	0
1.5112	1.7193	-0.7353	C	0	0	0	0	0	0	0	0	0	0	0	0
1.5161	2.7708	-1.5559	C	0	0	0	0	0	0	0	0	0	0	0	0
2.4232	3.3443	-1.7176	H	0	0	0	0	0	0	0	0	0	0	0	0
0.6169	3.0861	-2.0749	H	0	0	0	0	0	0	0	0	0	0	0	0
3.7799	2.1551	-0.1635	O	0	0	0	0	0	0	0	0	0	0	0	0
4.4657	1.9319	0.4901	H	0	0	0	0	0	0	0	0	0	0	0	0

0.2794	0.9296	-0.4802	C	0	0	0	0	0	0	0	0	0	0	0	0
0.1940	0.0754	0.3787	O	0	0	0	0	0	0	0	0	0	0	0	0
4.1545	-0.6609	1.8523	N	0	0	0	0	0	0	0	0	0	0	0	0
4.1674	-1.5051	2.7332	O	0	0	0	0	0	0	0	0	0	0	0	0
4.4707	0.5081	2.0391	O	0	0	0	0	0	0	0	0	0	0	0	0
-1.9608	0.5988	-1.0667	C	0	0	0	0	0	0	0	0	0	0	0	0
-2.3984	-0.3817	-1.9330	C	0	0	0	0	0	0	0	0	0	0	0	0
-2.7336	1.0071	0.0305	C	0	0	0	0	0	0	0	0	0	0	0	0
-3.6417	-0.9842	-1.7160	C	0	0	0	0	0	0	0	0	0	0	0	0
-1.7685	-0.6673	-2.7704	H	0	0	0	0	0	0	0	0	0	0	0	0
-3.9654	0.3952	0.2434	C	0	0	0	0	0	0	0	0	0	0	0	0
-4.4263	-0.6007	-0.6309	C	0	0	0	0	0	0	0	0	0	0	0	0
-3.9948	-1.7534	-2.3970	H	0	0	0	0	0	0	0	0	0	0	0	0
-4.5839	0.6826	1.0887	H	0	0	0	0	0	0	0	0	0	0	0	0
-0.7438	1.2427	-1.3066	O	0	0	0	0	0	0	0	0	0	0	0	0
-5.7701	-1.2490	-0.3633	C	0	0	0	0	0	0	0	0	0	0	0	0
-6.5512	-0.4802	-0.3228	H	0	0	0	0	0	0	0	0	0	0	0	0
-6.0066	-1.9216	-1.1960	H	0	0	0	0	0	0	0	0	0	0	0	0
-5.7691	-2.0202	0.9300	C	0	0	0	0	0	0	0	0	0	0	0	0
-5.0748	-2.8599	0.9859	H	0	0	0	0	0	0	0	0	0	0	0	0
-6.5348	-1.7231	1.9809	C	0	0	0	0	0	0	0	0	0	0	0	0
-7.2322	-0.8873	1.9537	H	0	0	0	0	0	0	0	0	0	0	0	0
-6.4929	-2.3052	2.8979	H	0	0	0	0	0	0	0	0	0	0	0	0
-2.1954	1.9839	0.8071	O	0	0	0	0	0	0	0	0	0	0	0	0
-2.9713	2.4384	1.9139	C	0	0	0	0	0	0	0	0	0	0	0	0
-3.1479	1.6236	2.6229	H	0	0	0	0	0	0	0	0	0	0	0	0
-2.3782	3.2194	2.3885	H	0	0	0	0	0	0	0	0	0	0	0	0
-3.9248	2.8527	1.5715	H	0	0	0	0	0	0	0	0	0	0	0	0

1	2	4	0	0	0	0
1	6	4	0	0	0	0
1	7	1	0	0	0	0
2	3	4	0	0	0	0
2	8	1	0	0	0	0
3	4	4	0	0	0	0
3	11	1	0	0	0	0
4	5	4	0	0	0	0
4	21	1	0	0	0	0
5	6	4	0	0	0	0
5	9	1	0	0	0	0
6	10	1	0	0	0	0
11	12	1	0	0	0	0
11	13	1	0	0	0	0
11	17	1	0	0	0	0
13	14	2	0	0	0	0
13	19	1	0	0	0	0
14	15	1	0	0	0	0
14	16	1	0	0	0	0
17	18	1	0	0	0	0
19	20	2	0	0	0	0
19	33	1	0	0	0	0
21	22	2	0	0	0	0
21	23	4	0	0	0	0

24 25 2 0 0 0 0
24 26 4 0 0 0 0
24 33 1 0 0 0 0
25 27 4 0 0 0 0
25 28 1 0 0 0 0
26 29 4 0 0 0 0
26 42 1 0 0 0 0
27 30 4 0 0 0 0
27 31 1 0 0 0 0
29 30 4 0 0 0 0
29 32 1 0 0 0 0
30 34 1 0 0 0 0
34 35 1 0 0 0 0
34 36 1 0 0 0 0
34 37 1 0 0 0 0
37 38 1 0 0 0 0
37 39 2 0 0 0 0
39 40 1 0 0 0 0
39 41 1 0 0 0 0
42 43 1 0 0 0 0
43 44 1 0 0 0 0
43 45 1 0 0 0 0
43 46 1 0 0 0 0
